# N-Cadherin Mediates Neuronal Cell Survival through Bim Down-Regulation

**DOI:** 10.1371/journal.pone.0033206

**Published:** 2012-03-12

**Authors:** Elise C. Lelièvre, Charlotte Plestant, Cécile Boscher, Emeline Wolff, René-Marc Mège, Hélène Birbes

**Affiliations:** 1 INSERM, UMRS-839, Paris, France; 2 Université Pierre et Marie Curie-Paris 6, Paris, France; 3 Institut du Fer à Moulin, Paris, France; University of Illinois at Chicago, United States of America

## Abstract

N-cadherin is a major adhesion molecule involved in the development and plasticity of the nervous system. N-cadherin-mediated cell adhesion regulates neuroepithelial cell polarity, neuronal precursor migration, growth cone migration and synaptic plasticity. In vitro, it has been involved in signaling events regulating processes such as cell mobility, proliferation and differentiation. N-cadherin has also been implicated in adhesion-dependent protection against apoptosis in non-neuronal cells. In this study, we investigated if the engagement of N-cadherin participates to the control of neuronal cells survival/death balance. We observed that plating either primary mouse spinal cord neurons or primary rat hippocampal neurons on N-cadherin recombinant substrate greatly enhances their survival compared to non-specific adhesion on poly-L-lysine. We show that N-cadherin engagement, in the absence of other survival factors (cell-matrix interactions and serum), protects GT1-7 neuronal cells against apoptosis. Using this cell line, we then searched for the signaling pathways involved in the survival effect of N-cadherin engagement. The PI3-kinase/Akt survival pathway and its downstream effector Bad are not involved, as no phosphorylation of Akt or Bad proteins in response to N-cadherin engagement was observed. In contrast, N-cadherin engagement activated the Erk1/2 MAP kinase pathway. Moreover, N-cadherin ligation mediated a 2-fold decrease in the level of the pro-apoptotic protein Bim-EL whereas the level of the anti-apoptotic protein Bcl-2 was unchanged. Inhibition of Mek1/2 kinases with U0126, and the resulting inhibition of Erk1/2 phosphorylation, induced the increase of both the level of Bim-EL and apoptosis of cells seeded on the N-cadherin substrate, suggesting that Erk phosphorylation is necessary for cell survival. Finally, the overexpression of a phosphorylation defective form of Bim-EL prevented N-cadherin-engagement induced cell survival. In conclusion, our results show that N-cadherin engagement mediates neuronal cell survival by enhancing the MAP kinase pathway and down-regulating the pro-apoptotic protein Bim-EL.

## Introduction

Cadherins are a family of transmembrane proteins that mediate calcium-dependent homophilic cell-cell contacts. They provide anchorage in between neighboring cells by interacting with the actin cytoskeleton through catenins [1]. Cadherins play crucial roles in embryonic morphogenesis by affecting cell shape, differentiation, migration and proliferation [2]. In addition, cadherin-mediated cell-cell interactions may regulate the balance between cell survival and cell death during development and maintenance of tissue homeostasis. Indeed several studies have reported that disruption of cadherin adhesion initiates apoptosis in various epithelial [3], [4], [5] and tumor cells [6], [7], [8], [9]. However, the mechanisms whereby cadherin adhesion contributes to cell fate by regulating the survival/cell death balance is poorly understood by comparison to the well-known molecular mechanism of anoïkis (apoptosis induced by the loss of cell contacts with the extracellular matrix (ECM)) [10]. Depending on cell types, the initiation and execution of anoïkis, which is refrained in physiological conditions by integrin engagement, is mediated by different pathways that all converge to the regulation of anti-apoptotic and pro-apoptotic Bcl-2 family proteins, leading to activation of caspases and subsequent activation of endonucleases, DNA fragmentation and eventually cell death [10].

Proteins of the Bcl-2 family are major regulators of apoptosis [11]. Anti-apoptotic members, such as Bcl-2, Bcl-xL, and Mcl-1 contain four Bcl-2 homology (BH) domains while the pro-apoptotic proteins fall into two categories: the Bax-like proteins, such as Bax and Bak that contain multiple BH domains, and the BH3-only members, Bad, Bid, Bmf, and Bim that contain only the BH3 domain. These pro-apoptotic proteins promote the release of apoptogenic factors from the mitochondria. Several studies indicate that the BH3-only members bind to the pro-survival Bcl-2 proteins and neutralize them, thereby allowing Bax-like proteins to initiate apoptosis [12]. The different BH3-only members respond to different forms of cellular stress and are subject to regulation at both transcriptional and posttranslation levels. During anoïkis, activation of MAP kinase, PI-3 kinase/Akt and JNK pathways regulate Bcl-2 family members such as the anti-apoptotic Bcl-2 and Bcl-xL proteins or the pro-apoptotic proteins Bad or Bim [10]. Bim primary transcripts undergo alternative splicing to generate three major isoforms: Bim-S, Bim-L and Bim-EL, the latter representing the predominant isoform in most tissues [13]. Bim-EL is up-regulated during anoïkis while its down-regulation by RNA interference inhibits anoïkis [14]. It has been established that Bim acts as a critical mediator of neuronal apoptosis [13], [15], [16], [17]. Indeed, Putcha et al., have reported that Nerve Growth Factor (NGF) deprivation in sympathetic neurons rapidly induced expression of Bim-EL [16]. Conversely, Bim deletion conferred protection against cytochrome c release and neuronal apoptosis upon NGF deprivation. Similar protective effect of Bim deletion against apoptosis was observed in cerebellar granule neurons subjected to K^+^ withdrawal [16].

N-cadherin is the predominant form of cadherin expressed ubiquitously in the early neural tube [18]. N-cadherin participates to the development and functional organization of the adult neural tissue [19]. This receptor is necessary for the polarisation of the neuroepithelial sheets, the radial organization of developing cortical plate and likely the migration of neuronal precursors [20]. It is also implicated in neurite outgrowth, dendritic arborization, axon guidance, and in the early stages of synaptogenesis [21]. Later in development, N-cadherin localizes at synapses [22], where it not only plays an adhesive role but also participates to the regulation of synaptic function and plasticity [23], [24], [25]. In this study, we assessed whether cadherin-mediated cell-cell adhesion provides a survival signal for neuronal cells and thereby focused our study on the contribution of N-cadherin. For that purpose, we used an immobilized N-cadherin (N-cad) recombinant protein coated on culture plates mimicking cell-cell contact formation to tightly control cadherin engagement [26], [27]. Controlled mobilization of N-cadherin was shown to induce cell cycle exit and myogenic differentiation [28] and to promote neurite outgrowth [29], [30]. Our work demonstrates that N-cadherin-mediated adhesion provides neuronal cells with pro-survival signal, protecting them from apoptosis in the absence of growth factors and extracellular matrix stimuli. In addition, we describe a molecular pathway by which cadherin regulates cell survival. Indeed, we report that the engagement of N-cadherin results in the phosphorylation of Erk1/2 and the down-regulation of the pro-apoptotic protein Bim, whereas the PI3-kinase/Akt survival pathway and Bcl-2 protein are not affected.

## Materials and Methods

### Antibodies and reagents

The following antibodies were used for western blotting : rabbit anti-Akt (1∶1000; Cell Signaling Technology, Beverly, MA), rabbit anti-phospho-Akt (1∶1000; Cell Signaling Technology), rabbit anti-Erk1/2 (1/10000; Upstate Biotechnology, Inc. Lake Placid, NY), mouse anti-phospho-Erk1/2 (1/10000; Sigma), mouse anti-Bcl-2 (1/500; Transduction Laboratories, Becton Dickinson Europe, Le Pont de Claix, France), rabbit anti-Bcl-xL (1/1000; Cell Signaling Technology), rabbit anti-Bim (1/1000; Pharmingen, San Diego, CA), rabbit anti-phospho-Bad (1/1000; Cell Signaling Technology), rabbit anti-PARP (1/1000; Cell signalling Technology), mouse anti-β-actin (1/20000; clone AC-15; Sigma), mouse anti-α-tubulin (1∶20000, clone E7, Developmental Studies Hybridoma Bank). Mouse anti-cytochrome c (1/200, clone 6H2.B4; Pharmingen) anti-βIII-tubulin (1/1000, clone Tuj1; Covance, CA) and anti-paxillin antibodies (1/100, Upstate Biotechnologies) monoclonal antibodies were used for immunostaining. 4′,6-diamino-2-phenylindole, dihydrochloride (DAPI) was from Molecular Probes (Eugene, OR). U0126 and LY294002 were from Cell Signaling Technology, PMA was from Sigma, Triton X-100 was from Pierce.

The expression vector encoding for the dominant negative mutant form of N-cadherin deleted of its extracellular domain (DN-Ncad), initially described in [31], has been modified by replacing the Myc tag by the DsRed protein coding sequence [32]. The expression vectors coding for wild type Bim-EL and the ERK-dependent phosphorylation site defective mutant Bim-EL (S69G) have been described in [33].

### Cell culture

Mouse GT1-7 cells [34] were grown at 37°C, 5%CO_2_, in Dulbecco's Modified Eagle Medium with 4.5 g/l glucose (DMEM, Life Technologies) supplemented with 10% fetal bovine serum (FBS, Life Technologies) and antibiotics (100 IU/ml penicillin and 100 µg/ml streptomycin, Life Technologies). GT1-7 cells were transfected using Lipofectamine 2000 (Life Technologies). Cells were seeded at 8.10^4^ cells/cm^2^ and transfected 24 hours later according to manufacturer's instructions.

Primary mouse ventral spinal cord and rat hippocampal neurons were prepared as previously described [35], [36]. Briefly, ventral spinal cord of E12.5 mouse embryos from the OF1 pregnant mice were isolated and digested with trypsin for 10 min at 37°C in Ham F10 modified medium containing 0.025% trypsin. Tissues were then mechanically dissociated in L15 medium containing DNAse I and the supernatant was laid on a BSA 4% solution and centrifuged. Spinal cells were seeded on coated coverslips at 2.10^4^ cells/cm^2^ in Neurobasal medium containing 0.5 mM L-glutamine and cultivated for 24 hours to 48 hours at 37°C, 5% CO_2_. Hippocampi from E18.5 rat embryos (Sprague Dawley pregnant rat, R. Janvier laboratories) were isolated in HBSS-20 mM Hepes and digested with trypsin 0,05% 10 min at 37°C, then mechanically dissociated. Hippocampal cells were recovered in MEM medium supplemented with 2 mM glutamine, 1 mM sodium pyruvate and seeded on coated coverslips at 2.10^4^ cells/cm^2^ and cultivated for 24 hours at 37°C, 5% CO_2_. All experiments with animals were in accordance with the guidelines of the French Agriculture and Forestry Ministry for handling animals (decree 87849, license 75–765-Renouvellement) and approved by the “Charles Darwin ethical committee in animal experimentation, Paris” under permit number Ce5/2010/064.

### Substrates coating

Adhesive substrates were prepared as described in [28]. Briefly, glass coverslips or thermosterilized bacterial 35-mm Petri dishes (Falcon, Becton Dickinson Europe) were incubated with goat anti-mouse Fcγ fragment antibody (Jackson ImmunoResearch, West Grove, PA) at 1 µg/cm^2^ in 0.1 M borate buffer pH 8.0 overnight at 4°C. Plates were washed and then incubated with purified Ncad-Fc chimera (extracellular domain of the chicken N-cadherin fused to the mouse IgG2b Fc fragment [27]) at a concentration of 1 µg/cm^2^ for 2 hours at room temperature. For the control samples, glass coverslips or dishes were incubated with poly-L-lysine (PL, 0.01%, Sigma) or fibronectin (FN, 10 µg/cm^2^, Invitrogen), in water overnight at 4°C. After washes, surfaces were then saturated with 1.5% purified BSA (Sigma) in PBS for 5–10 min at room temperature.

### Controlled cell adhesion assays

Specific cell adhesion and spreading on coated surfaces were performed according to [28]. Briefly, to preserve cell-surface cadherins, twenty-four hours serum-starved GT1-7 cells were mechanical dissociated in trypsin-free conditions with PBS, 3.5 mM EDTA, 1.5% BSA on ice. Cells were then plated on the different adhesion substrates in serum-free conditions to prevent growth factors-mediated survival and at low cell density (8.10^4^ cells/cm^2^) to prevent cell-cell contacts between neighboring cells. For experiments using inhibitors, cells were incubated 1 hour with the specific inhibitor prior to mechanically dissociation and throughout culture on the different substrates with inhibitors at the concentrations and times indicated. To induce Bad and Erk1/2 protein phosphorylation, confluent cells were treated with Phorbol 12-myristate 13-acetate (PMA) at 10 ng/ml for 1 hour.

### Calcium switch assays

Confluent GT1-7 cell monolayers were deprived of serum for 24 hours, and the N-cadherin-mediated cell-cell contacts were disrupted by treatment with 4 mM EGTA in DMEM for 40 min at 37°C. The calcium-free medium was removed and N-cadherin contacts were allowed to re-establish by addition of DMEM, containing 1.8 mM Ca^2+^. The time of re-addition of Ca^2+^ was considered as 0 min. Following calcium restoration at 37°C, cells were harvested, lysed, protein extracts separated by SDS-PAGE and immunoblotted as described below.

Alternatively, serum deprived GT1-7 cells were maintained in suspension in 1% agarose coated 60 mm culture dishes (10.10^4^ cells/dish) to prevent cell attachment. Cells were either incubated in the presence of 1.8 mM Ca^2+^ (provided by the DMEM medium) or in the presence of DMEM plus 2.5 mM EDTA (0 mM free Ca^2+^) to prevent cadherin-dependent cell-cell adhesion. At various time points, cells were washed once with cold PBS, collected and processed for analysis of PARP cleavage by immunoblotting.

### Western blotting

Cells were lysed in RIPA modified buffer (50 mM Tris-HCl, pH 7.4, 1% NP-40, 0.25% DOC, 150 mM NaCl, 20 mM sodium pyrophosphate, 50 mM sodium fluoride, 1 mM sodium orthovanadate and protease inhibitor cocktail (Roche)). Lysates were vortexed 20 min at 4°C and then cleared by centrifugation at 10, 000 rpm for 15 min at 4°C. Equal amounts of proteins (20 µg determined by micro-BCA kit, Pierce) were loaded and separated by SDS-PAGE. After transfer at 4°C, nitrocellulose membranes were blocked with 5% nonfat milk in Tris-buffered saline, pH 8.0, containing 0.1% Tween 20 (TBST) prior to addition of the corresponding primary antibody and followed with IRDye-coupled secondary antibodies (Rockland) against rabbit or mouse immunoglobulins. For detection of phosphorylated proteins, antigens were detected with phospho-specific antibodies diluted in 5% BSA in TBST at 4°C. Protein bands were identified with Odyssey Imaging System (LI-COR Biosciences). Signals were quantified with LI-COR software. The membranes were reprobed with an anti-β-tubulin, anti-β-actin monoclonal or anti-pan Erk1/2 polyclonal antibodies for normalization.

### Immunocytochemistry and TUNEL assay

Cytochrome c release was followed by immunostaining as described previously [37]. Briefly, at the end of the treatment time, cells were washed gently once with PBS followed by fixation in 4% formaldehyde for 15 min. The fixed cells were washed three times with PBS 5 min each, followed by permeabilization in 0.15% Triton X-100 in PBS for 15 min. The cells were then blocked for 60 min in blocking buffer (2% BSA in PBS) followed by 4 hours incubation with a mouse monoclonal antibody against cytochrome c. The cells were washed three times for 10 min each in blocking buffer followed by 1 hour incubation with a rhodamine-labeled goat anti-mouse IgG (1/500, Jackson Immunology). The cells were washed three times for 10 min in PBS, counterstained with 0.5–1 µg/ml DAPI, to examine nuclear morphology and quantify apoptosis. TUNEL assay was performed using the In Situ Cell Death Detection Kit, TMR Red (Roche Diagnostics) following the manufacturer's protocol. Cells were viewed under a fluorescence microscope (Leica DM6000) and images were captured with a digital camera using Metamorph software. Different fields (at least 200 cells) were counted for each experiment.

### Statistical analysis

Data were compared by ANOVA followed by protected *t* test for multiple comparisons. Paired sample means were compared using the *t* test. *P* values of 0.05 or less were considered statistically significant.

## Results

### N-cadherin engagement promotes survival of primary neurons

In primary neuron culture studies, most culture protocols have been optimized to grow neurons on poly-ornithine (PO) or poly-L-lysine (PL) attachment factors. Associated culture media have been adapted by adding sera and/or B27 supplement. We observed that when these media additives were omitted from E18.5 rat hippocampus neurons cultured on PL, although cells initially bound to the plate, they rapidly died (Fig. S1). In contrast, replacing PL by recombinant Ncad-Fc (N-cad) as a substrate allowed maintaining healthy neurons in culture for at least 24 to 48 hours (Fig. S1). Although cells were seeded in similar conditions for each substratum, the observed reduction in healthy neurons on PL might be due to a deficit in the initial adhesion of these cells on PL. To assess this point, we counted the actual number of cells attached at 1 hour, 4 and 24 hours after seeding, and observed that there was no deficit of cell binding on PL compared to N-cad at early time points (Fig. S2A). This was confirmed by the fact that we were able to observe by time-lapse microscopy that cells initially attach then die (data not shown).

**Figure 1:**
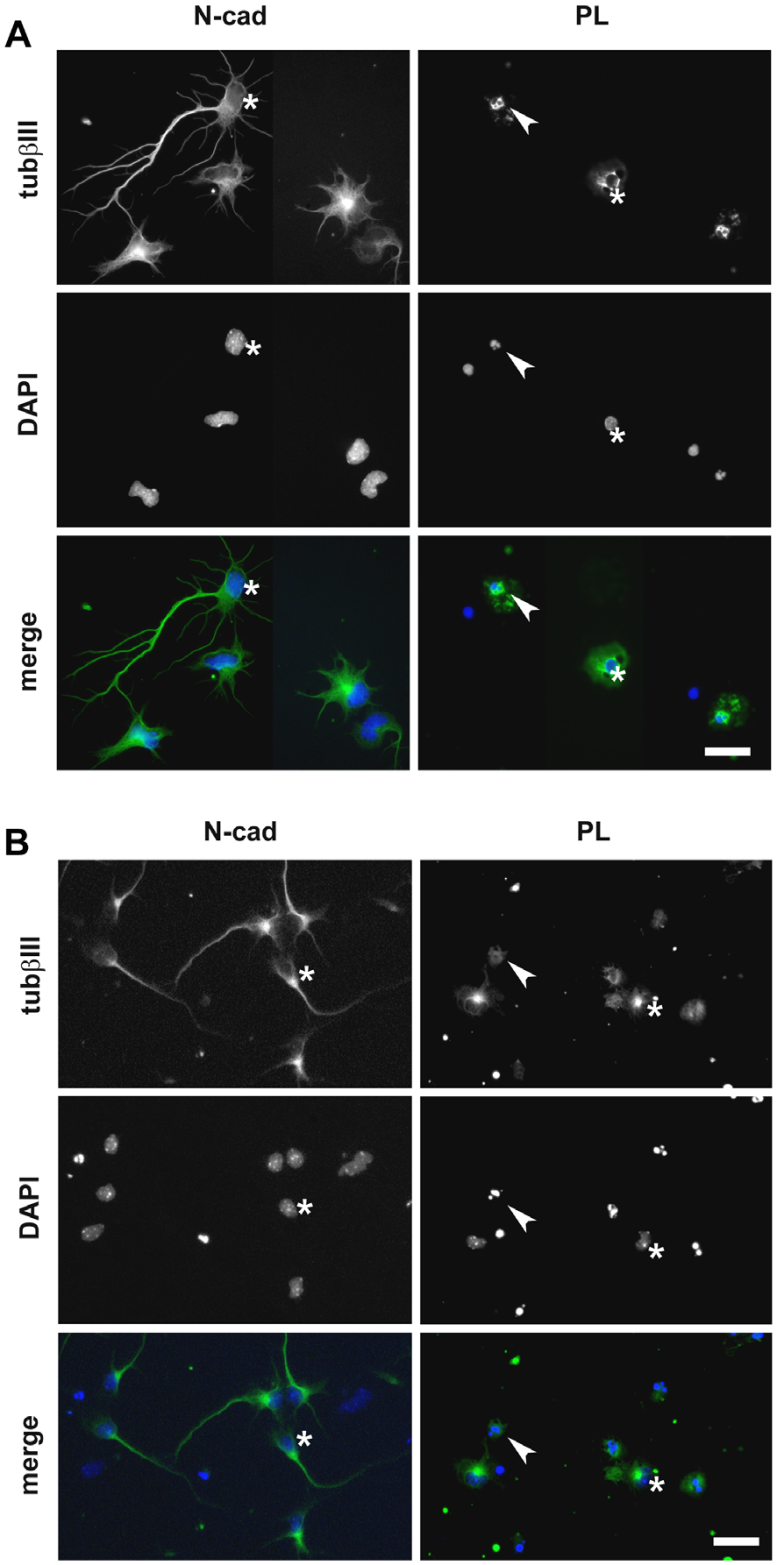
N-cadherin engagement sustains spinal cord and hippocampal neuronal cell survival in minimal medium conditions. Neurons were dissociated from E18.5 rat hippocampus (A) or E12.5 mouse ventral spinal cords (B) and cultured on N-cad or PL in MEM and Neurobasal basal medium, for hippocampal and spinal neurons, respectively. After 24 hours, they were fixed, and stained with anti-βIII tubulin antibody to identify neurons and with DAPI to assess the nuclear morphology. Arrows point toward cells dying with condensed nuclei while asterisks indicate cells with healthy nuclei. Scale bar: 10 µm in A; 20 µm in B.

**Figure 2:**
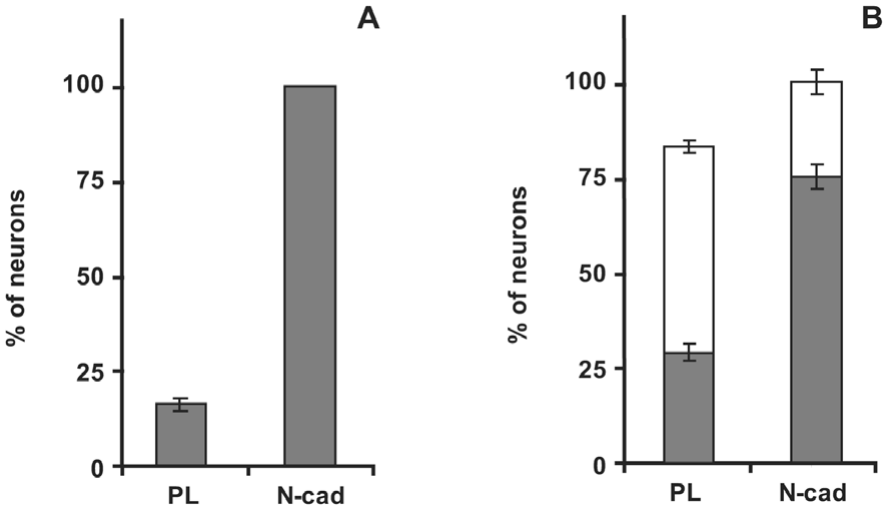
N-cadherin engagement sustains spinal cord and hippocampal neuronal cell survival. (A) In 24 hours hippocampal cultures, the number of βIII tubulin-positive cells per µm^2^ were counted on each substrate and expressed as a percentage of that on N-cad substrates, arbitrarily fixed to 100%. (B) The numbers of βIII tubulin-positive cells with condensed (white bars) or non-condensed nuclei (dark bars) were determined in ventral spinal cords cultures after 24 hours on each substrate. The number of βIII tubulin-positive cells per µm^2^ was expressed as a percentage of the number of βIII tubulin-positive cells per µm^2^ on N-cad substrates, arbitrarily fixed to 100%. Results are expressed as the mean ± SD of two independent experiments.

To further characterize the effect of N-cadherin on survival of rat hippocampal neurons in the absence of B27 additive, cultures were fixed after 24 hours and stained for βIII-tubulin to identify neuronal cells. The total number of remaining βIII-tubulin positive cells was dramatically lower on the PL substrate (below 20%) in comparison to the number of cells remaining on the N-cad substrate (Fig. 1A, 2A), confirming that in these basal conditions, N-cad had a survival effect on primary hippocampal neurons. Similar observations were made for primary murine spinal cord cultures (Fig. 1B, 2B). In this case, the difference in the total number of βIII-tubulin positive cells between the PL and N-cad substrate was not as dramatic after 24 hours. However, DAPI staining revealed that the percentage of presumably apoptotic condensed nuclei was higher on PL (54±2% of βIII-tubulin positive cells with condensed nuclei) than on N-cad substrates (25±3% of βIII-tubulin positive cells with condensed nuclei). In addition, when we analyzed cell death by terminal deoxynucleotidyl transferase dUTP nick end labeling (TUNEL) we observed that the number of TUNEL positive cells in hippocampal cultures increased with time on PL whereas it remained very low on N-cad (Fig. S2, B). Altogether these observations show that primary neurons die, likely by apoptosis, in these stringent conditions when plated on PL, while they displayed a high survival rate when plated on N-cad. They suggest thus that N-cadherin engagement promotes their survival.

### N-cadherin engagement protects GT1-7 cells from undergoing apoptosis

However, molecular and biochemical studies on primary culture models are difficult to perform due to the limited amount of biological material. Therefore to study the molecular pathway whereby N-cadherin mediates cell survival, we decided to use GT1-7 immortalized hypothalamic neurons [34], which express N-cadherin. These cells usually grow as confluent layers in serum-supplemented media which provides them cell-cell adhesion as well as diffusive (growth factors) and anchorage-dependent (fibronectin/vitronectin) survival factors.

**Figure 3:**
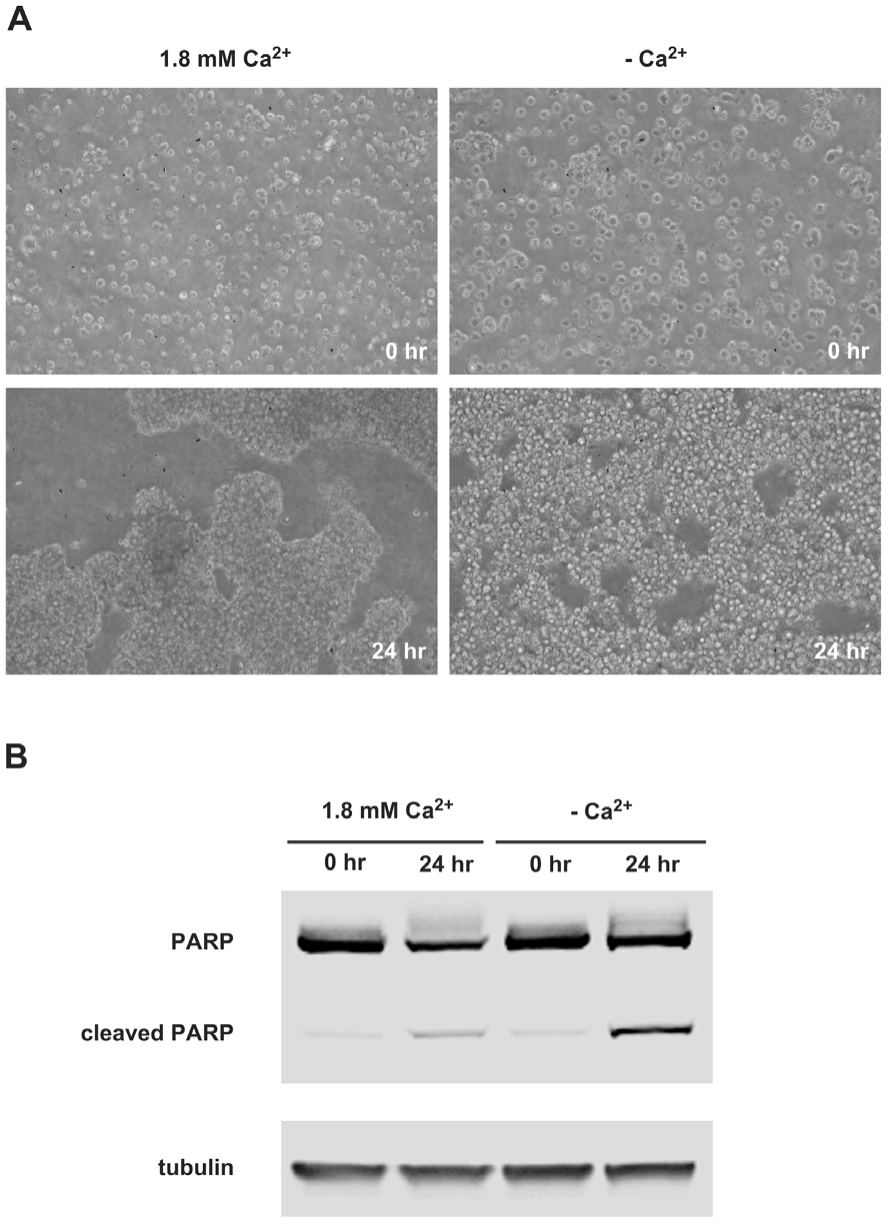
Inhibition of calcium-dependent cell-cell adhesion induces apoptosis of GT1-7 cells in suspension. Serum-starved GT1-7 cells were cultured in suspension in agarose-coated dishes in the presence of calcium (1.8 mM) or without calcium (2.5 mM EDTA). (A) Phase-contrast photomicrographs were taken at time 0 and 24 hr of incubation. (B) Cells lysates were then analyzed by western blotting for PARP cleavage. Western blot representative of two independent experiments.

To investigate the contribution of cadherin-mediated cell-cell adhesion to neuronal cell survival, we forced serum-starved GT1-7 cells to grow in suspension by plating them on agarose-coated dishes, as previously reported [7], [8]. We then manipulated extracellular calcium concentrations to allow (1.8 mM Ca^2+^) or inhibit (0 mM free Ca^2+^) calcium–dependent cell-cell adhesion. The presence of a physiological concentration of Ca^2+^ induced the expected cell aggregation, while GT1-7 cells only loosely aggregated in the absence of free Ca^2+^ consistent with a massive inhibition of cell-cell adhesion (Fig. 3A). To determine the involvement of calcium-dependent cell adhesion on cell survival, we then analyzed the cleavage of poly (ADP-ribose) polymerase (PARP) induced in apoptotic cells by activated caspases. The inhibition of calcium-dependent cell-cell adhesion specifically induced the PARP cleavage whereas PARP cleavage remained very low in cells cultured in the presence of calcium (Fig. 3B). These results show that cells forced to grow under serum-free anchorage-independent conditions survive as multicellular aggregates and that Ca^2+^-dependent cell adhesion sustain cell survival in these conditions, as previously reported in other cellular models [7], [8].

Next, we set up more controlled cell culture conditions to investigate specifically the effect of N-cadherin adhesion on cell survival. GT1-7 cells were seeded either on N-cad substrate to specifically engage N-cadherin homophilic liganding or on PL to deprive them of specific adhesion, in (i) serum-free conditions to exclude cell-ECM and growth factors activated signaling, and (ii) at low density to prevent uncontrolled cell-cell contact formation (Fig. S3). In some control experiments, cells were seeded on fibronectin substrate (FN) to allow for controlled integrin mobilization, a situation known to mediate cell survival.

**Figure 4:**
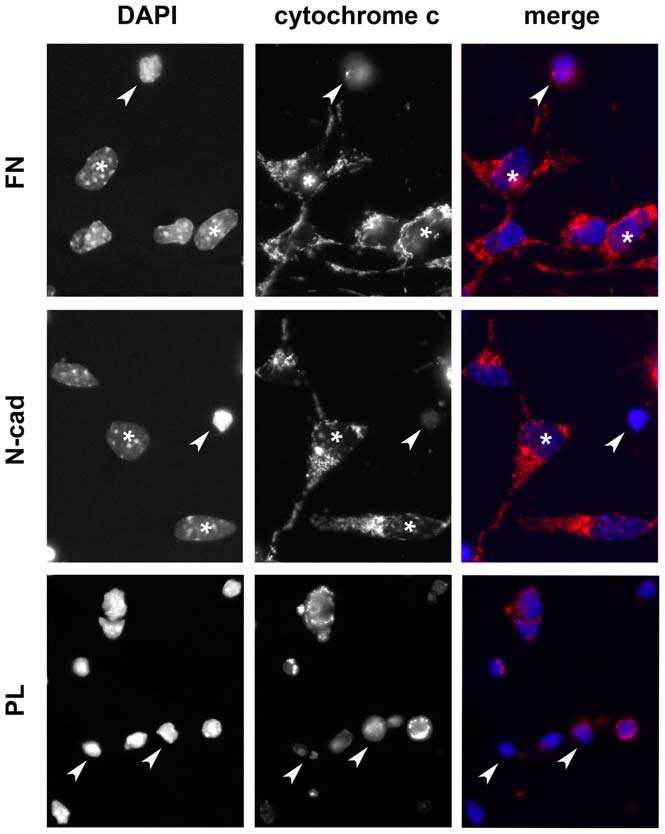
N-cadherin engagement protects neuronal cells from nuclear condensation and cytochrome c release. Serum-starved GT1-7 cells were seeded at low density on FN, N-cad or PL substrates and grown in serum-free medium. After 24 hours, cells were fixed and immunostained for cytochrome c (right) while nuclei were stained with DAPI (left). Arrows point toward cells showing condensed nuclei and diffuse or absence of cytochrome staining while asterisks indicate cells with healthy nuclei and mitochondrial cytochrome staining. Scale bar: 20 µm.

**Figure 5:**
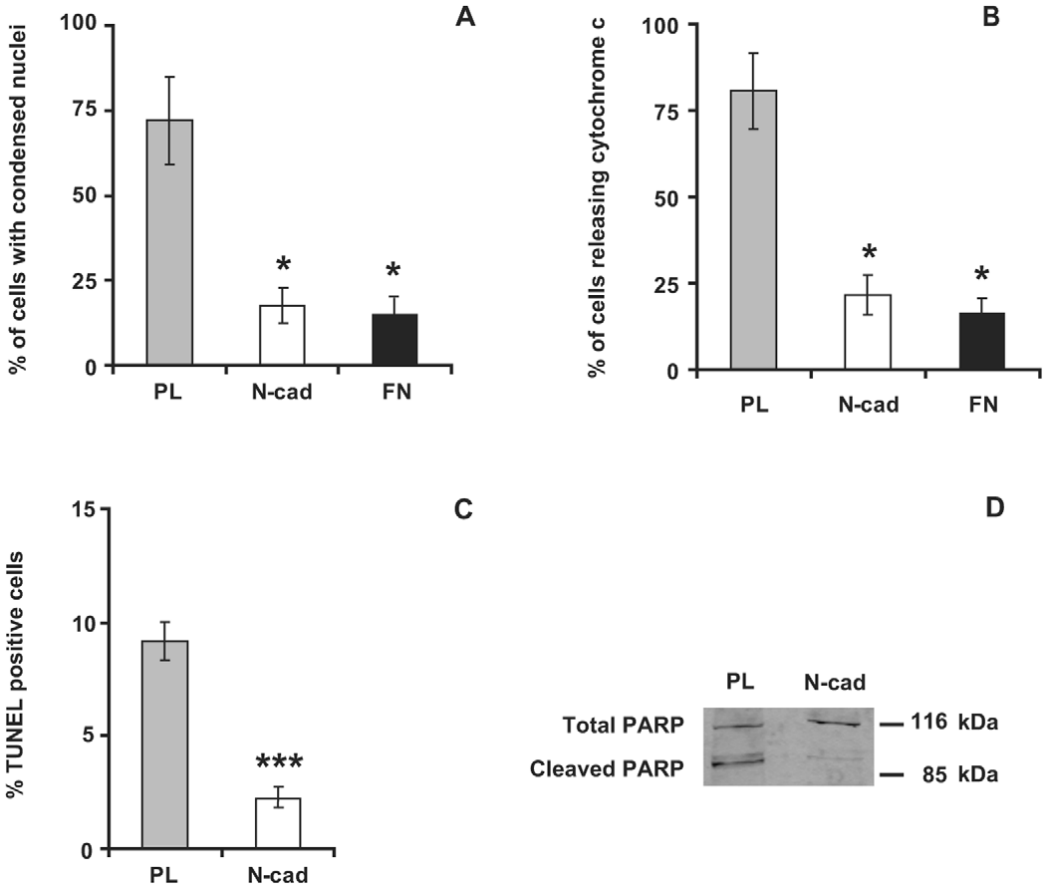
N-cadherin engagement protects neuronal cells from apoptosis. GT1-7 cells were cultured as indicated in Figure S2 for 24 (A, B) or 4 hours (C) and processed for nuclear condensation (A), cytochrome c release (B) as well TUNEL libeling (C). PARP cleavage was analyzed by western blotting of equivalent protein content from cell lysates (D). Results in A, B and C are expressed as the mean ± SD of four independent experiments. Western blotting in D is representative of three independent experiments. Asterisks (*) or (***) indicate a significant difference (P<0.05) or (P<0.01) respectively as compared with PL.

GT1-7 cell death was first evaluated by the nuclear condensation after DNA staining (Fig. 4). 72% of cells seeded on PL were positive for nuclear condensation whereas only 18% of cells seeded on N-cad exhibited condensed nuclei after 24 hours (Fig. 5A). Interestingly, a similar low proportion of condensed nuclei (∼20%) was also obtained among cells seeded on fibronectin substrate (FN), where cell-matrix-anchorage was expected to induce an integrin-mediated cell survival pathway. To exclude the possibility that cell survival observed on N-cad was the consequence of integrin engagement induced by the deposition of ECM components by GT1-7 cells themselves, we evaluated integrin pathway activation by phosphorylated FAK and paxillin immunostaining. We did not detect paxillin (Fig. S4) nor phosphorylated forms of FAK (data not shown) accumulation at the ventral face of the cells seeded on PL or N-cad while both stainings were strongly accumulated at focal adhesions in GT1-7 cells seeded on FN (Fig. S4 and data not shown). To confirm that the survival effect was the result of a specific engagement of N-cadherin, we tested the ability of a dominant negative mutant form of N-cadherin [32] to perturb cell survival triggered by N-cadherin. This DN-Ncad mutant was expressed in GT1-7 cells that were then seeded on N-cad substrate. While most of the control GT1-7 cells transfected with a GFP-encoding plasmid displayed healthy nuclei morphology after 24 hours on the N-cad substrate, more than 50% of the DsRed positive cells displayed condensed nuclei (Fig. S5), indicating that the expression of DN-Ncad counteracts the protective effect of the N-cad substrate. These results suggest that N-cadherin engagement protects cells against apoptosis in a specific manner.

To gain insight on the specific cell death pathway activated, cytochrome c release from mitochondria to the cytosol was evaluated by immunostaining (Fig. 4). After 24 hours, a vast majority of cells (80%) seeded on PL showed a cytochrome c distribution characteristic of cytosolic localization and very often a total loss of cytochrome c, as reported in [38], (Fig. 5B). In contrast, most of the cells seeded on N-cad showed a punctuated or tubular perinuclear pattern characteristic of the mitochondria network and only 22% of the cells had released their cytochrome c out of mitochondria. Similar results were obtained with cells seeded on FN (positive control of cell survival), with less than 20% of cells showing diffuse cytochrome c staining (Fig. 5B). Time course studies showed that nuclear condensation and cytochrome c release were already high 6 hours after plating cells on PL whereas the percentage of apoptotic figures remained low and constant for cells on N-cad (Fig. S6).

To confirm that these cytological changes were part of an ongoing apoptotic process that was repressed by N-cadherin engagement, we analyzed cell death by two complementary approaches: (i) TUNEL staining and (ii) PARP cleavage. After 4 hours of culture, the number of TUNEL positive GT1-7 cells was significantly higher on PL (8.6±0.9%) than on N-cad (2.1±0.6%) indicating that the deprivation of specific cell adhesion (PL substrate) induced apoptotic cell death (Fig. 5C). Moreover, when GT1-7 cells were seeded on PL, PARP cleavage was detected by the appearance of the characteristic 85 kDa fragment. By contrast, cells seeded on N-cad showed a reduced PARP cleavage (Fig. 5D). Altogether, these results indicate that N-cadherin engagement is sufficient to sustain GT1-7 cell survival by protecting them from apoptotic cell death.

### PI3-kinase/Akt pathway is not involved in N-cadherin engagement

**Figure 6:**
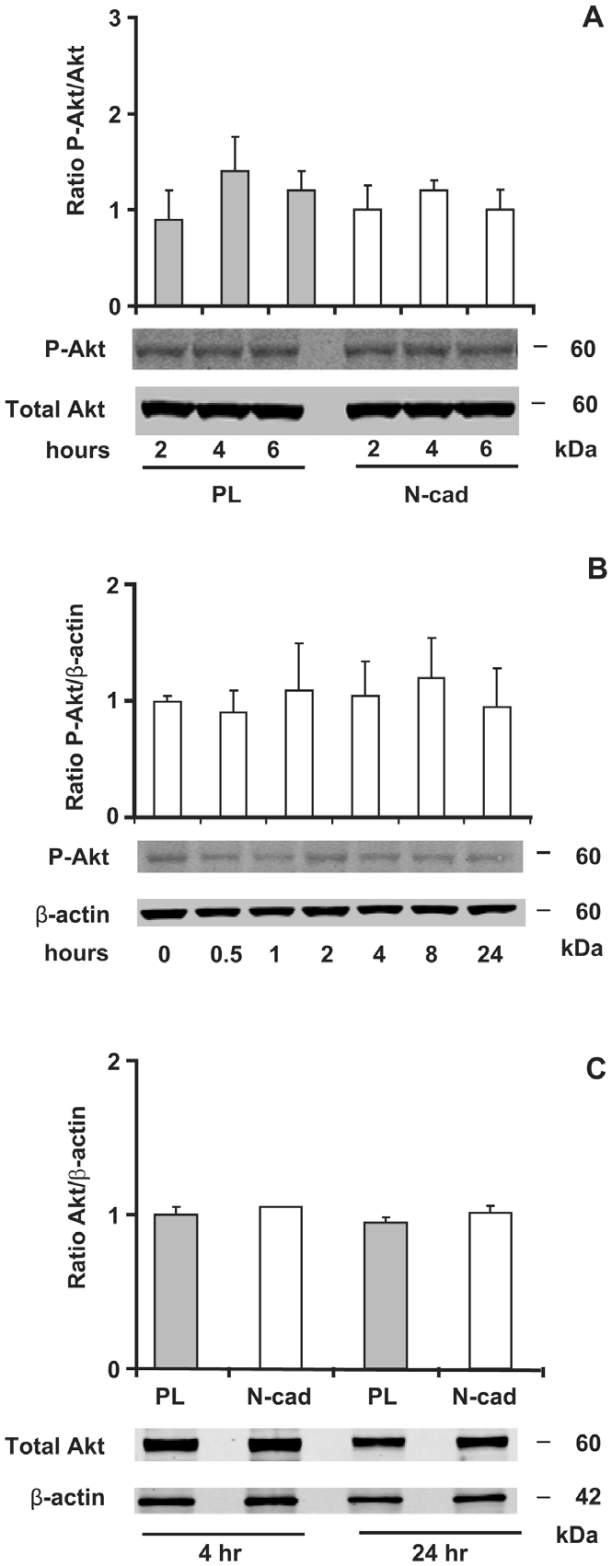
Akt is not involved in N-cadherin-mediated GT1-7 cell survival. Serum-starved cells were seeded at low density on PL or N-cad and grown for the indicated time in serum-free medium (A, C). In (B), confluent cell cultures were subjected to a calcium switch (see “Experimental Procedures”). Equivalent amount of cell lysates was separated on 4–12% SDS-PAGE, transferred onto nitrocellulose membranes, and immunobloted with antibodies to phospho-specific Ser473 Akt, total Akt and β-actin. The relative densities of phospho-Akt were normalized to total Akt, and the relatives densities of Akt were normalized to β-actin. Results are expressed as the mean ± SD of three independent experiments.

To elucidate the pathways involved in the protective effects of N-cadherin mobilization, we studied the activation of the PI3-kinase/Akt pathway, a key regulator of cell survival. First, we investigated whether N-cadherin mobilization could activate Akt kinase by phosphorylation on Ser-473. We previously showed that cells require at least 2 hours in order to spread on N-cadherin substrate; therefore, Akt phosphorylation was investigated by western blotting analysis using a phospho-specific antibody starting at 2 hours after plating. As cell death had reached a plateau at 6 hours, phosphorylation studies were conducted up to 6 hours. Spreading of cells on N-cad did not affect Akt phosphorylation at any time point studied (Fig. 6A). To confirm these results, we used a calcium switch approach with GT1-7 grown at confluency. Although less specific than Ncad-Fc mediated engagement, calcium restoration rapidly initiates cadherin-dependent cell-cell contact formation [39] allowing study of earlier time points. However, Akt phosphorylation was not changed even at earlier time points (Fig. 6B), confirming that Akt phosphorylation was not activated by N-cadherin engagement.

Alternatively, Akt could be a direct substrate for caspases. Indeed, it has been shown that Akt is cleaved by caspases leading to a reduction in Akt protein level in MDCK cells detached from the ECM. In turn this cleavage of Akt contributes to anoïkis [40]. To test this hypothesis, we assessed the total amount of Akt at various time points, but cellular levels of total Akt protein were not decreased on PL substrate compared with those on N-cad (Fig. 6C). Altogether, these observations suggest that the PI3-kinase/Akt pathway is not involved in the anti-apoptotic effect of N-cadherin in GT1-7 cells. In addition, the PI3-kinase inhibitor LY294002 had no effect on cell survival, nuclear condensation and TUNEL labeling of cell seeded on N-cad (data not shown). Thus, we investigated the involvement of other pathways, such as MAP kinase signaling, which has been reported to mediate cell survival [10].

### MAP kinase Erk signaling is required for cell survival induced by N-cadherin engagement

**Figure 7:**
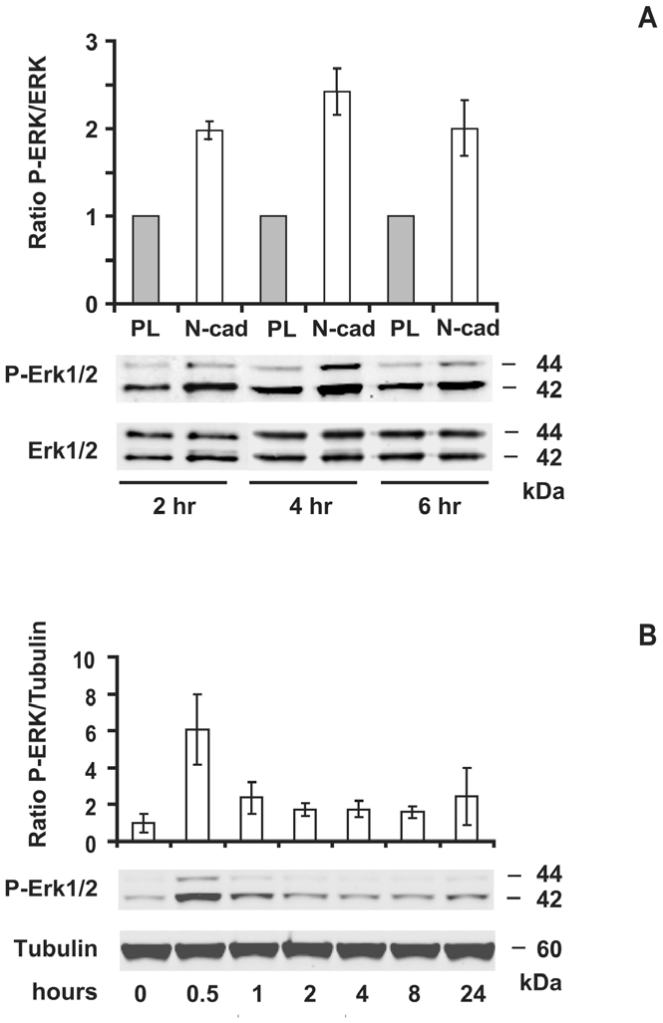
Erk1/2 phosphorylation is up-regulated by N-cadherin engagement. Serum-starved GT1-7 cells were seeded at low density on PL or N-cad and grown for the indicated time in serum-free medium (A); alternatively confluent GT1-7 cell cultures were subjected to a calcium switch (B). Equivalent amount of proteins were blotted onto nitrocellulose membranes that were incubated either with anti-phospho Erk1/2, anti-total Erk1/2, or anti-α-tubulin. The relative densities of phospho-Erk1/2 were normalized to total Erk1/2 (A) or to α-tubulin (B). Histograms present the data of three independent experiments (mean ± SD).

To examine whether N-cadherin engagement could trigger a signal transduction pathway involving the MAP kinases, we evaluated the phosphorylation state of Erk1 and Erk2 using a phospho-specific antibody. The phosphorylation of Erk1/2 was increased in a time-dependent manner in cells seeded on N-cad substrate as compared to cells seeded on PL. Phosphorylation of Erk1 and Erk2 was maximal at 4 hours with a 2.5 fold increase compared to cells seeded on PL (Fig. 7A). Furthermore, the restoration of intercellular adhesion in the calcium switch assay induced a strong elevation of Erk1 and Erk2 phosphorylation with a 6-fold increase observed at 30 min (Fig. 7B). These results support an activation of the Erk1/2 pathway in response to N-cadherin engagement in accordance with our previous report on the activation of Erk1/2 by N-cadherin engagement in primary spinal cord neurons.

**Figure 8:**
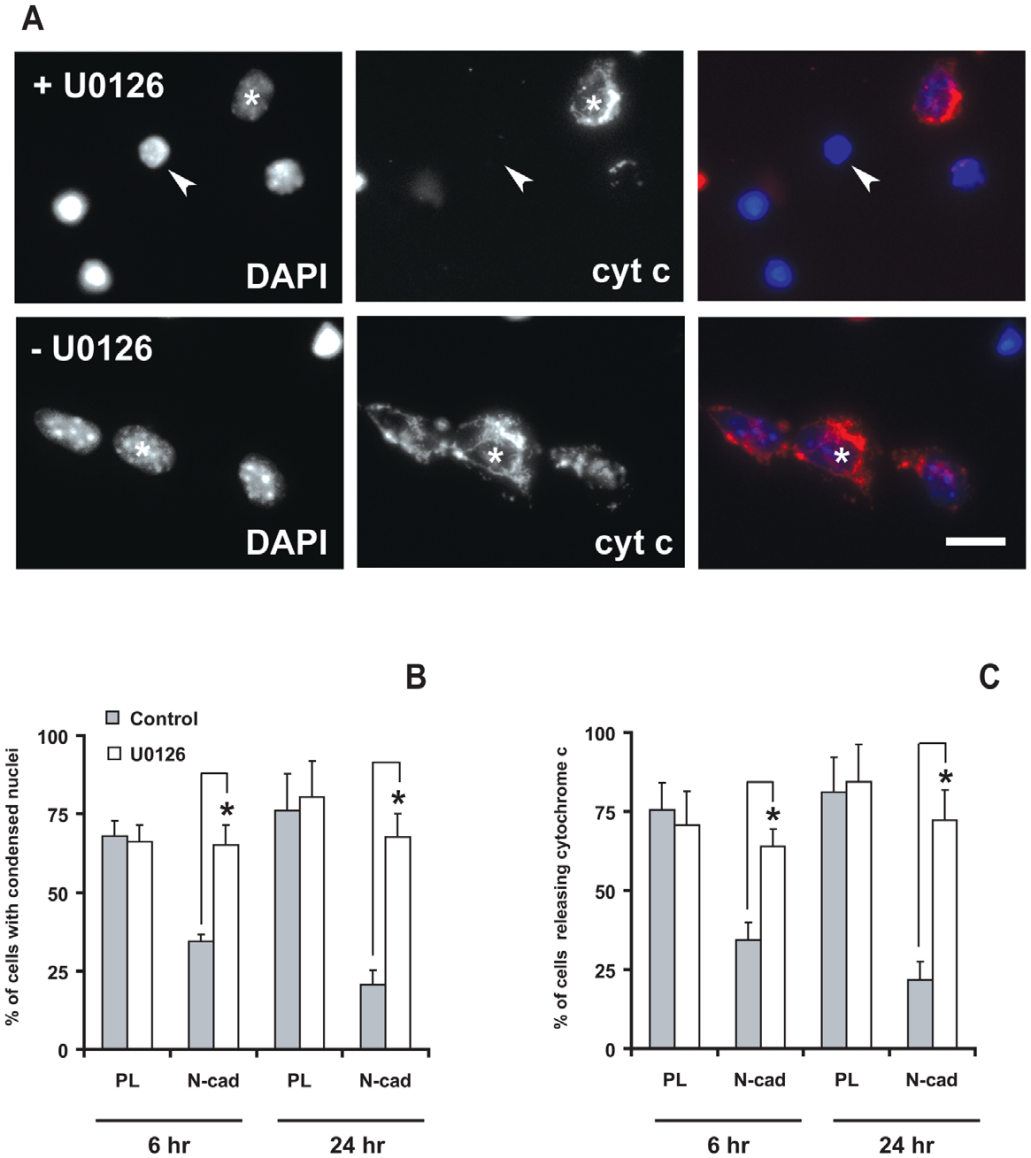
Mek 1/2 inhibitor induces apoptosis on cells seeded on N-cadherin. Serum-starved GT1-7 cells were pretreated with 20 µM U0126 or vehicle for 1 hour, then seeded at medium density on N-cad with or without 20 µM U0126 for 6 hours (A). Apoptosis was evaluated by determination of nuclei condensation following DAPI staining and cytochrome c release following immunostaining. Arrows point toward cells showing condensed nuclei and absence of cytochrome c staining while asterisks indicate a cell with healthy nuclei and cytochrome c staining. Scale bar: 20 µm. (B, C). The percentage of cells with condensed nuclei and diffuse/absent cytochrome c labeling was quantified at 6 and 24 hours in treated and non-treated cells seeded on PL or N-cad. Results are expressed as the mean ± SD of four independent experiments. Asterisks indicate a significant difference (P<0.05) as compared with PL.

To confirm the involvement of the MAP kinase pathway in relaying the anti-apoptotic signal triggered by N-cadherin engagement, we examined the effect of U0126, a specific inhibitor of the Mek1/2 kinase activity. We used this inhibitor at 20 µM. Indeed this concentration was sufficient to abolish Erk1/2 phosphorylation during the calcium switch (Fig. S7). U0126-treated cells were seeded either on PL or N-cad and apoptotic cells were scored at 6 and 24 hours using nuclear condensation and cytochrome c release markers (Fig. 8). U0126 treatment did not affect cell death on PL. However, this treatment reversed the protective effects of N-cadherin since a similar apoptosis level as for cells seeded on PL was observed on the Ncad substrate (Fig. 8B, C). These results indicate that N-cadherin protection is lost in the presence of Mek1/2 inhibitor and support the hypothesis that the Erk1/2 pathway is involved cell survival downstream of N-cadherin engagement.

### N-cadherin engagement does not regulate Bcl-2 or Bad protein levels

**Figure 9:**
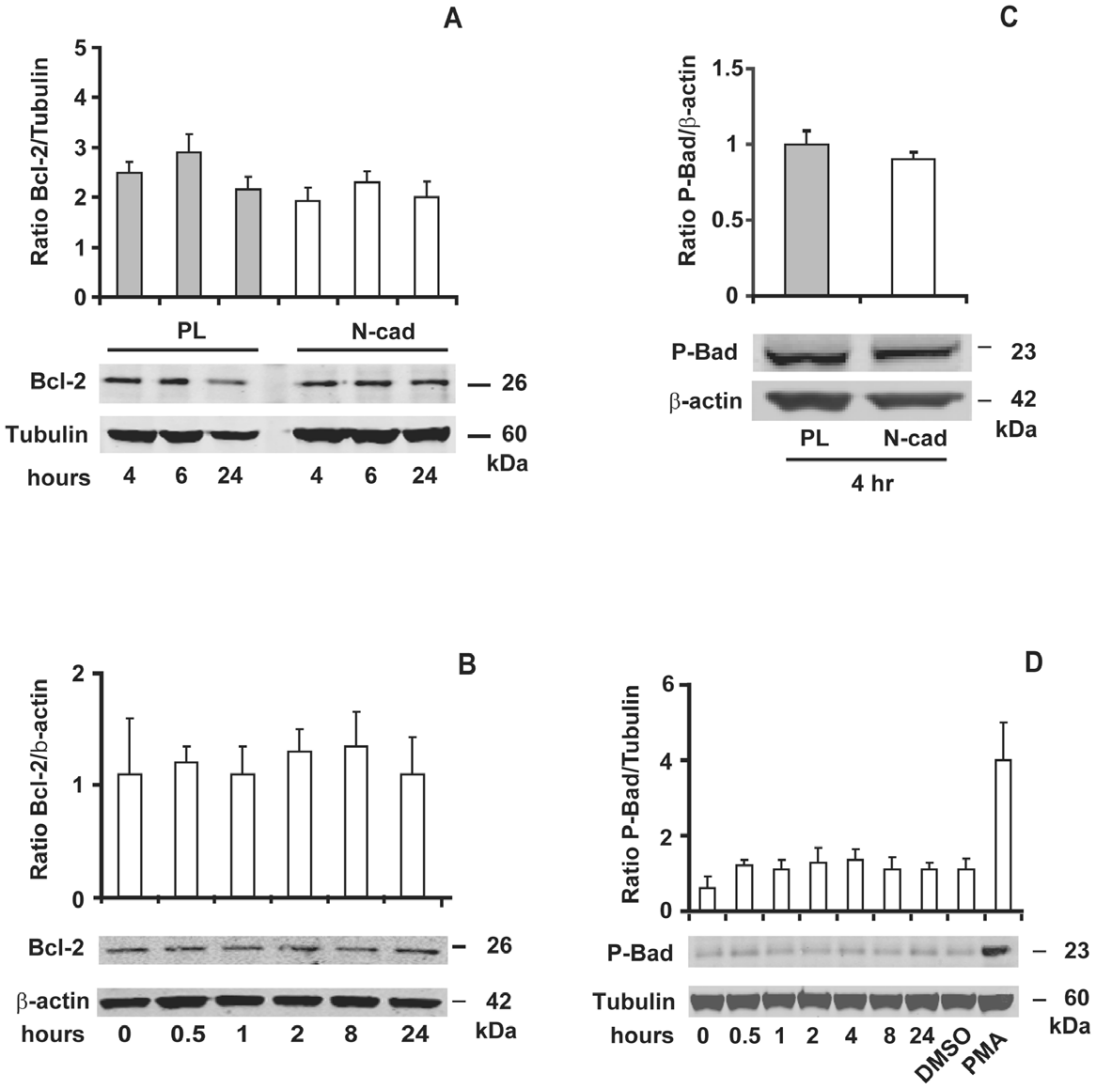
N-cadherin engagement does not affect Bcl-2 protein level and Bad phosphorylation. Serum-starved GT1-7 cells were seeded at low density on PL or N-cad and grown for the indicated time (A) or for 4 hours (C) in serum-free medium. In B-D, cells were subjected to a calcium switch protocol. As a positive control, cells were treated with 10 ng/ml of PMA or DMSO for 1 hour. Equivalent amount of proteins were immunobloted with antibodies to Bcl-2 and phospho-Bad. Relative densities of Bcl-2 and phospho-Bad were normalized with either α-tubulin, or β-actin. Results are the mean ± SD of three independent experiments.

We next aimed at determining the downstream targets of the MAP kinases involved in cell survival pathway induced by the N-cadherin engagement. Several studies have reported that, during anoïkis, Bcl-2 family members such as the anti-apoptotic Bcl-2 and Bcl-xL proteins or the pro-apoptotic proteins Bad or Bim are regulated by activated MAP Kinases [10]. Therefore, we measured the Bcl-2 protein levels by immunoblotting at several time points. Bcl-2 protein levels remained constant after N-cadherin engagement and at levels equivalent to those of cells seeded on PL (Fig. 9A). Consistent results were obtained with the calcium switch assay (Fig. 9B), suggesting that cell survival induced by N-cadherin engagement was not mediated by an increase of the anti-apoptotic Bcl-2 protein levels.

It has also been demonstrated that the early phosphorylation of the pro-apoptotic protein Bad on Ser-112 by MAP kinases leads to its inactivation [41] resulting in cell survival. However, we did not detect any increase of Bad phosphorylation in cells seeded on N-cad at the earlier time point (4 hours) as compared to cells seeded on PL (Fig. 9C). Moreover, calcium restoration in confluent GT1-7 cells did not induce Bad phosphorylation at any time point tested (Fig. 9D). These results indicate that Bcl-2 and Bad proteins take not part in the survival pathway induced by N-cadherin mobilization.

### N-cadherin engagement down-regulates the pro-apoptotic protein Bim-EL

**Figure 10:**
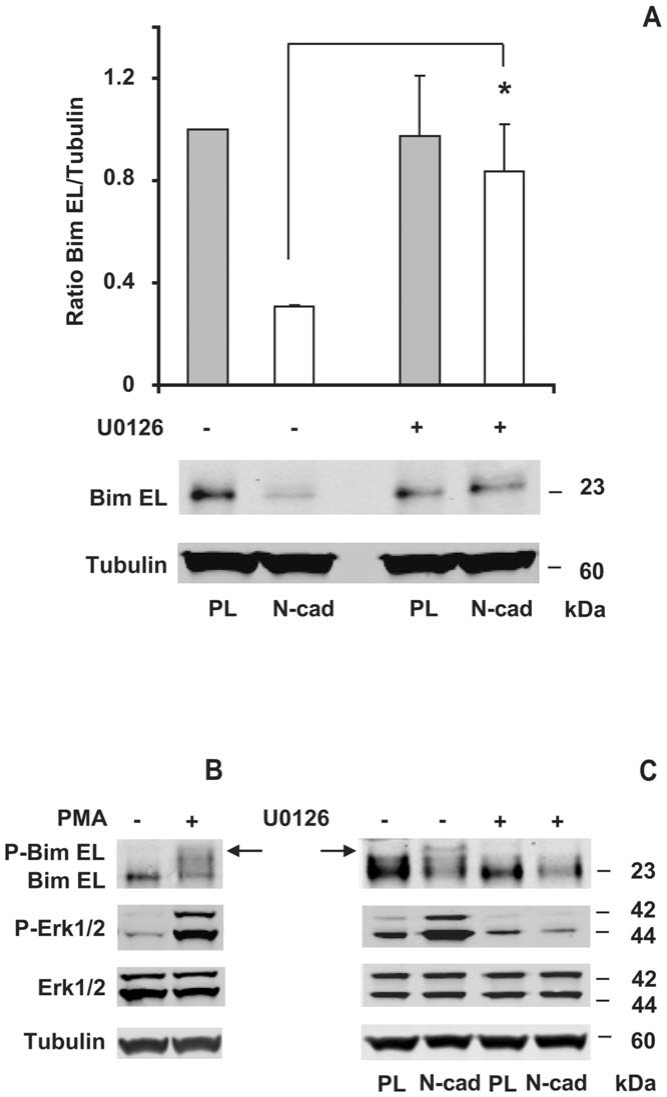
N-cadherin engagement down-regulates Bim-EL protein levels in a Mek1/2 dependent manner. Serum-starved cells were seeded at medium density on PL or N-cad and incubated with or without 20 µM U0126 for 24 hours (A) or 4 hours (C), then protein were extracted and analyzed by western blotting with either anti-Bim, anti-phospho-Erk1/2, anti-Erk1/2, or anti α-tubulin antibodies. Relative densities of Bim-EL were normalized to α-tubulin. As a positive control of Bim-EL mobility shift, adherent cells were treated with 10 ng/ml of PMA for 1 hour (B). Represented data are the mean of three independent experiments. Asterisks indicate a significant difference (P<0.05) as compared with no treatment. Arrows show Bim-EL mobility shift.

The Bim protein is another critical regulator of apoptosis downstream of MAP kinase signaling. We determined whether N-cadherin engagement affects Bim protein levels. In confluent GT1-7 cells, Bim was expressed at low levels and Bim-EL was the only form of Bim detected. However in cells deprived of cell-matrix and cell-cell contacts (PL substrate) Bim-EL was up-regulated, while it remained low in cells seeded on N-cad. At 24 hours Bim-EL protein level was 3.5 fold higher in cells seeded on PL than on cells seeded on N-cad (Fig. 10A), suggesting that N-cadherin engagement might favor cell survival by down-regulating Bim-EL. To ascertain whether Erk1/2 pathway acts downstream of N-cadherin to regulate the level of Bim-EL protein, N-cad seeded cells were treated with 20 µM U0126. This treatment induced a 3 fold increase in Bim-EL levels thereby reaching similar levels to those in cells seeded on PL (Fig. 10A). The increase of this pro-apoptotic protein was consistent with the raise of the apoptotic index in cells seeded on N-cad and treated with U0126 (Fig. 8). Altogether these findings indicate that MAP kinase activation following N-cadherin engagement could sustain GT1-7 cell survival by maintaining Bim-EL protein levels low. Interestingly, we observed that adhesion of GT1-7 cells to the pro-survival FN substrate down-regulates Bim-EL in a similar Erk1/2-dependent manner than N-cadherin (Fig. S8). Luciano et al. showed that phosphorylation of Bim-EL by Erk1/2 on Ser-69 triggers its degradation by the proteasome pathway and promotes cell survival [33]. We thus searched for changes in the status of Bim-EL phosphorylation. As a positive control of Bim-EL phosphorylation, cells were treated with PMA. Addition of PMA induced a mobility shift of Bim-EL and was correlated with the expected phosphorylation of Erk1/2 (Fig. 10B). This mobility shift of Bim-EL observed in GT1-7 cells was quite similar to the one observed by Luciano et al. in different cell lines and tissues as a result of Bim-EL phosphorylation [33]. At early time points (4 hours), we detected the same mobility shift of the Bim-EL protein in cells spread on N-cad which was abrogated by U0126 treatment (Fig. 10C). These results suggest that upon N-cadherin engagement, Bim-EL is phosphorylated by Erk1/2 kinases.

### Overexpression of a S69G phosphorylation defective form of Bim-EL prevents N-cadherin cell survival

**Figure 11:**
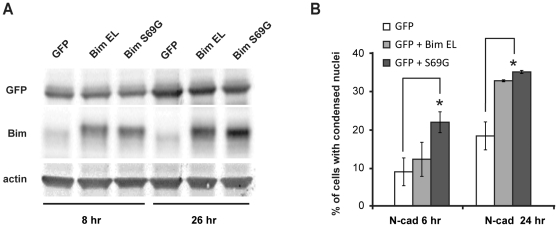
Overexpression of Bim-EL S69G prevents N-cadherin induced cell survival. Expression vectors coding for wild type Bim-EL and Bim-EL S69G were lipofected in 24 hours starved GT1-7 cells together with pEGFP at a 2/1 ratio. Cell lysates of equivalent protein content were analyzed by western blotting at 8 hours and 26 hours post-transfection for Bim and GFP expression; actin was analyzed as loading control (A). Two hours after lipofection, cells were harvested from transfected cultures and seeded on N-cadherin. Preparations were fixed 6 hours and 24 hours later (corresponding to 8 hours and 26 hours post-transfection) and nuclei stained. The percentage of GFP positive cells with condensed nuclei was then determined for each condition in duplicates (≥100 GFP^+^ cells counted each time). Represented data are the mean of two independent experiments. Asterisks indicate a significant difference (P<0.05) as compared with GFP expression alone.

In order to test whether the phosphorylation and down-regulation of Bim-EL was involved in mediating N-cadherin engagement-induced cell survival, we looked at the effect of overexpression of an Erk-dependent phosphorylation site defective mutant of Bim-EL (Bim-EL S69G) [33] on the survival of GT1 cells seeded on N-cadherin (Fig. 11). To prevent massive accumulation of the pro-apoptotic protein in cells before their settlement on N-cadherin, Bim-EL expression vector were lipofected 2 hours before plating on the N-cadherin substrate. To identify transfected cells, the Bim-EL vector was lipofected together with a GFP expressing vector. We first assessed the expression of Bim-EL S69G protein in confluent GT1-7 cells 8 hours and 24 hours post-transfection (Fig. 11A). While low levels of endogenous Bim-EL were detected in GFP alone transfected cells, a 2.5 to 3 fold increase in Bim expression was observed in Bim-EL S69G transfected cell cultures, for a percentage of transfected cells around 10% of the cell culture population, indicating that the Bim-EL mutated protein indeed highly accumulates in individual transfected cells.

We then analyzed the effect of Bim-EL S69G overexpression on the survival of cells seeded on N-cadherin by determining the percentage of transfected cells with a condensed nucleus 6 hours and 24 hours after plating (Fig. 11B). The expression of GFP alone did not lead to an increase in the occurrence of dying cells compared to un-transfected cells plated on N-cadherin (compare with Figure 5). In contrast, the overexpression of Bim-EL S69G induced a significant increase in the number of apoptotic cells both at 6 and 24 hours. The overexpression of the wild type form of Bim-EL expressed at similar levels (Fig. 11A) had no significant effect on GT1-7 cells plated on N-cadherin at 6 hours, although it impaired the survival at 24 hours (Fig. 11B), suggesting that the accumulation with time of wild time Bim-EL may also lead to apoptosis due to the overwhelming of the Bim-EL phosphorylation and degradation pathway. Altogether, these data indicate that the accumulation of Bim-EL and in particular of its Erk- phosphorylation defective mutant (S69G) prevents N-cadherin-induced cell survival, supporting the hypothesis that N-cadherin triggers neuronal cell survival through a Bim-EL down-regulation.

## Discussion

**Figure 12:**
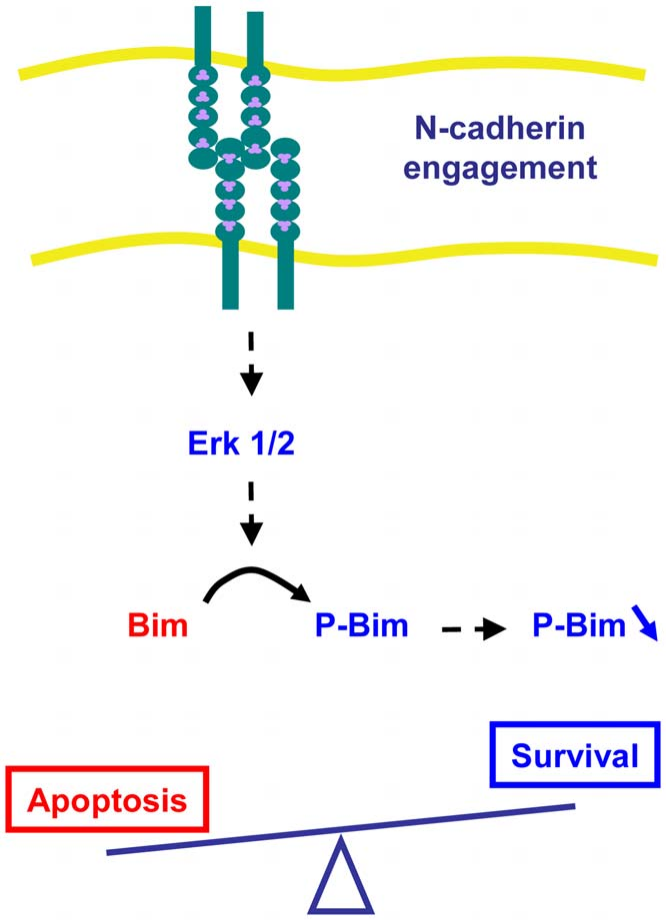
Hypothetical model for N-cadherin mediated cell survival signaling in neurons.

In this study, we accumulate convergent arguments to support the hypothesis that N-cadherin engagement provides neuronal cells with a pro-survival signal. We cannot formally exclude the contribution of other factors or adhesion molecules to this process. However, the anti-apoptotic effect of N-cadherin engagement was observed in tightly controlled conditions, that is in isolated cells, in the absence of growth factors, and in the absence of detectable integrin activation in focal adhesion complexes. Furthermore the survival effect of the N-cad substrate was alleviated by the expression of a dominant negative form of N-cadherin. These results strongly support the hypothesis that N-cadherin initiates an anti-apoptotic pathway in neurons and neuronal cells that is sufficient to sustain cell survival when they are deprived of other survival factors such as integrin interactions with the ECM. We further report that N-cadherin engagement mediates cell survival by enhancing the Erk1/2 kinase pathway and down-regulating the pro-apoptotic protein Bim-EL (Fig. 12). Bim-EL has been extensively involved in apoptotic pathways in neurons upon NGF deprivation or after K^+^ withdrawal [16]. Thus, we propose that N-cadherin engagement may antagonize a common apoptotic pathway activated in neurons upon various stress conditions.

The involvement of cadherins in cell survival has been described previously in different cell types either normal or tumoral [3], [7], [42], [43], [44]. However, the molecular mechanism whereby cadherin adhesion contributes to cell survival is still poorly understood and controversial. N-cadherin engagement has been shown to promote survival through the activation of the PI3- kinase/Akt and subsequent phosphorylation of the pro-apoptotic protein Bad in metastatic cancer cells [7] and in vein smooth muscle cells [45]. The PI3-kinase pathway has also been implicated in E-cadherin-mediated cell survival in Ewing Tumor cells [8] and in epithelial cells [4], [5]. Therefore, we examined the role of PI3-kinase/Akt pathway. N-cadherin ligation did not affect Akt and Bad phosphorylation. This was observed at different time points from 30 min to 24 hours and using two different approaches, calcium switch assay and biomimetic substrates. In addition, the inhibitor of PI3-kinase, LY294002, did not block the GT1-7 cell survival after N-cadherin engagement. In agreement with our results, Skaper et al. showed that the inhibition of PI3-kinase/Akt pathway failed to block the survival of hippocampal and cerebellar granule neurons induced by N-cadherin agonist [46]. N- and E-cadherin engagements have been shown to up-regulate Bcl-2 protein [7], [8], [42]. However, in our model, the levels of Bcl-2 protein were not affected by N-cadherin engagement. These results suggest that signaling pathways that affect cell survival in response to N-cadherin engagement in neuronal cells are independent of the PI3-kinase and Bcl-2 proteins and may involve alternative pathways such as MAP kinases.

Among the MAP kinases, Erk1 and 2 have been widely associated with neuronal cell survival, while JNK and p38 are often implicated in cell death [47]. Several studies suggest that Erk1/2 mediates neuroprotective activity of extracellular factors such as integrins and neurotrophins, as well as during survival of damaged neurons [5], [48]. We show that N-cadherin adhesion induces an increase of Erk1/2 phosphorylation and a subsequent inhibition of GT1-7 cell death. Pharmacological inhibition of the MAP kinase pathway using U0126 resulted in the inhibition of Erk1/2 phosphorylation and an increased number of apoptotic cells on N-cadherin. Therefore our results suggest that N-cadherin protects neurons against cell death by activating specifically Erk1/2. Similar observations were reported in ovarian carcinoma cells [49] and in epithelial cells [50] where E-cadherin engagement activates the Ras/Erk1/2 cascade but not the PI3-kinase/Akt pathway.

One critical regulator of apoptosis and target of the MAP kinase pathway is the pro-apoptotic protein Bim. The regulation of Bim activity is complex, involving both transcriptional and posttranscriptional mechanisms. Bim is normally expressed at low levels, and its pro-apoptotic activity kept in check by rapid phosphorylation by Erk1/2 resulting in its ubiquitination and proteosomal degradation. Reginato et al. have shown that attached MCF-10A cells contain low levels of Bim-EL and undetectable levels of Bim-L and Bim-S. All three isoforms were highly up-regulated after cell detachment from the ECM [14]. Fukazawa et al. reported that Bim-EL is the only form expressed in human HBC4 and MDA-MB231 cancer cell lines [51]. They showed that treatment with MAP Kinase inhibitors, which prone cells for apoptosis in the absence of cell anchorage, induced a reduction of Bim-EL phosphorylation together with an increase of Bim-EL protein levels. Similarly, we found that Bim-EL was the only form of Bim expressed, albeit at low level, in GT1-7 cells. Upon cell deprivation in cell-matrix and cell-cell contacts (PL substrate) Bim-EL was up-regulated. Conversely, N-cadherin engagement down-regulated Bim-EL protein levels in association with its phosphorylation as revealed by mobility shift [33]. Moreover, the Mek1/2 inhibitor U0126 prevented this phosphorylation, indicating that Bim-EL undergoes Erk1/2 dependent phosphorylation upon N-cadherin engagement, which may be responsible for its down-regulation. Interestingly, plating GT1-7 cells on fibronectin also elicited the down-regulation of Bim-EL, suggesting that integrin-mediated inhibition of anoikis may similarly rely, to some extent, on a Bim-EL down-regulation in neuronal cells as previously described in MCF-10A cells [14].

The MAP kinase pathway has been implicated in the regulation of Bim-EL expression in various other cell types like hematopoietic cells [52], fibroblasts [53], epithelial cells [14] and neurons [54]. Studies in PC12 neural cells have shown that NGF protects neurons against apoptosis by acute Mek1/2-dependent phosphorylation of Bim-EL and subsequent down-regulation of this specific isoform [54]. In epithelial [14] and cancer cells lines [51], inhibition of the MAP Kinase pathway was also reported to abolish Bim-EL phosphorylation and upregulate Bim protein levels in association with anoïkis. Phosphorylation of Bim-EL by Erk1/2 on Ser-69 has been reported to promote its degradation via the proteasome pathway [33], [55], [56], [57]. However, in our model, we were not able to detect an accumulation of Bim-EL upon treatment with proteasome inhibitors, neither we were able to detect Bim-EL ubiquitination (data not shown). Recently, an alternative mechanism was proposed by Ewings et al [58]. Erk1/2-dependent phosphorylation of Bim-EL promotes its rapid dissociation from Bcl-xL and Mcl-1 proteins and this dissociation may contribute to Bim-EL degradation [58]. Therefore, it remains to elucidate whether, in neuronal cells, Bim-EL phosphorylation drives its subsequent degradation and by which mechanisms. Nevertheless, in support to en essential role of Erk-dependent Bim-EL phosphorylation in triggering N-cadherin engagement-mediated neuronal cell survival, we observed an increased apoptosis on N-cadherin of cells overexpressing Bim-EL S69G. Interestingly, the overexpression of the wild type form of Bim-EL also impaired with a delay the survival of GT1-7 cells plated on N-cadherin. Altogether, these data indicate that the accumulation of Bim-EL prevents N-cadherin-induced cell survival, supporting the hypothesis that N-cadherin triggers neuronal cell survival through Bim-EL down-regulation.

It has long been recognized that cadherin function in close cooperation with, and organize, the cytoskeleton (for review: [1]). In turn, cytoskeleton and in particular microtubules may serve as scaffolds for signaling molecules that regulate apoptosis, such as Bim. It has been reported that Bim binds to the dynein light chain LC8 and is sequestered to the microtubule-associated dynein motor complex. Under apoptotic stimuli, Bim may be released and neutralizes Bcl-2 proteins [59]. A potential link between cadherins and microtubules has been postulated (for review: [60]). Therefore, it will be interesting to study if N-cadherin engagement affects Bim association with microtubules and whether this process is affected by Mek1/2-dependent Bim phosphorylation, although the quality of the available immunological reagents did not allowed us to test this hypothesis.

In conclusion, this study proposes a novel role of cell-cell contacts mediated by N-cadherin in signaling neuronal survival and provides insight into the underlying molecular mechanisms. It identifies MAP kinase activation and subsequent down-regulation of the pro-apoptotic protein Bim as a major signaling pathway in this process. To our knowledge, this is the first report that N-cadherin engagement mediates neuronal cell survival. The fact the GT1-7 cells response to cadherin engagement is shared with embryonic spinal cord and hippocampal neurons strongly support the hypothesis that promotion of neuronal survival by N-cadherin engagement is a largely spread regulation process. We cannot presently assign the relative contribution of this cell-cell contact signaling and the well-known contribution of cell-matrix adhesion and growth factors signaling in neuronal cell survival. However N-cadherin is a major mediator of cell-cell interactions during the development of the nervous system. Thus, our observations should have potential relevance for the control of the survival/death balance of neurons throughout development and plasticity of the mammalian nervous system.

## Supporting Information

Figure S1 **Culture of primary neurons in serum free non supplemented medium affects cell survival.** E18.5 rat hippocampus neurons were cultured in MEM medium in the presence of B27 additive and serum (+B27) either on PL (poly-L-lysine) or N-cad (immobilized Ncad-Fc). Alternatively they were cultured on the same types of substrate but in the absence of serum and B27 (−B27). After 24 hours, cells were fixed and stained with anti-βIII tubulin antibody to identify neurons. Although neurons grew well on PL as well as on N-cad in the presence of B27, they died on PL in the absence of the additive and serum. In addition, neurite extension was greatly increased on N-cad compared to PL even in the presence of B27 as expected from previous reports. Similar observations were made with cells cultured from 12.5 mouse ventral spinal cords (not shown). Scale bar: 20 µm.
(TIF)

Figure S2 **Compared adhesion/survival of primary hippocampal neurons on PL versus Ncad-Fc substrates.** E18.5 rat hippocampus neurons were cultured in MEM medium either on PL or N-cad in the absence of serum and B27 additive as reported in Figure 1A. Cultures were fixed at 1, 4 and 24 hours post-seeding and adherent cells were counted (A). Alternatively, preparations were processed for TUNEL labeling and the percentage of TUNEL positive cells determined (B).
(TIF)

Figure S3 **Experimental set up for controlled N-cadherin engagement.** In regular cell culture conditions (upper left), cells adhere to the substratum via adsorbed fibronectin (FN) and vitronectin (VN) provided by the serum (cell matrix adhesion) and adhere to each other via cadherins and other cell adhesion molecules (CAMs, cell-cell adhesion). To specifically activate either N-cadherin or fibronectin, GT1-7 cells were plated at low density on N-cad or FN, respectively. To deprive cells of specific cell adhesion, they were seeded on PL which mediates electrostatic cell-surface adhesion.
(TIF)

Figure S4 **Spreading of cells on N-cad does not mobilize the integrin pathway.** GT1-7 cells were cultured on N-cad for 4 hours, then fixed and fluorescently stained for F-actin with phalloïdin (red) and with anti-paxillin antibodies (green). Paxillin staining remained diffuse in the cytoplasm of these cells indicating that integrins were not mobilized on this substrate. In contrast, when GT1-7 cells were cultured on fibronectin (FN) for 4 hours, they displayed expected stress fibers and focal adhesions in which both stainings were strongly accumulated, indicated that indeed integrins have been mobilized and activated in focal adhesions in these culture conditions. Scale bars: 20 µm.
(TIF)

Figure S5 **Overexpression of a dominant negative form of N-cadherin drastically impairs the protective effect of N-cad on GT1-7 cells.** GT1-7 cells were lipofected with either a dominant negative form of N-cadherin fused to DsRed (DN-Ncad), or GFP alone, starved for 24 hours, then spread on the N-cad substrate. After 24 hours, cells were fixed and processed for nuclei staining. The percentage of transfected cells with normal and condensed nuclei was determined for each condition in three independent experiments and given as mean ± SD.
(TIF)

Figure S6 **Time course of GT1-7 cell death.** Serum-starved cells were seeded at low density on PL or N-cad and grown for the indicated time in serum-free medium. Cell death was measured by nuclear condensation (**A**) and cytochrome c release (**B**). Asterisks indicate a significant difference (P<0.05) as compared with PL.
(TIF)

Figure S7 **Dose dependent action of U0126 inhibitor.** Serum starved GT1-7 cells were treated (I) or not (C) with the U0126 inhibitor. Cultures were then subject to the calcium switch protocol and harvested just after (0′) or 30 minutes (30′) after calcium restoration. Proteins were extracted and analyzed for P-Erk1/2 content by western blotting. The results of two independent experiments are shown. The tubulin content in the extracts was used as a gel loading control.
(TIF)

Figure S8 **Spreading of GT1-7 cells on fibronectin also down-regulates Bim-EL protein levels.** Serum-starved cells were seeded at medium density on PL or FN and incubated with or without 20 µM U0126 for 24 hours. Equal amount of proteins were immunoblotted with either anti-Bim or anti-α-tubulin antibodies. Relative densities of Bim-EL were normalized to α-tubulin.
(TIF)
