# Supplementary figures and images for: N-Cadherin Mediates Neuronal Cell Survival through Bim Down-Regulation
Source: PLoS One. 2012 Mar 12;7(3):e33206. doi: 10.1371/journal.pone.0033206 (PMC3299760; doi:10.1371/journal.pone.0033206)

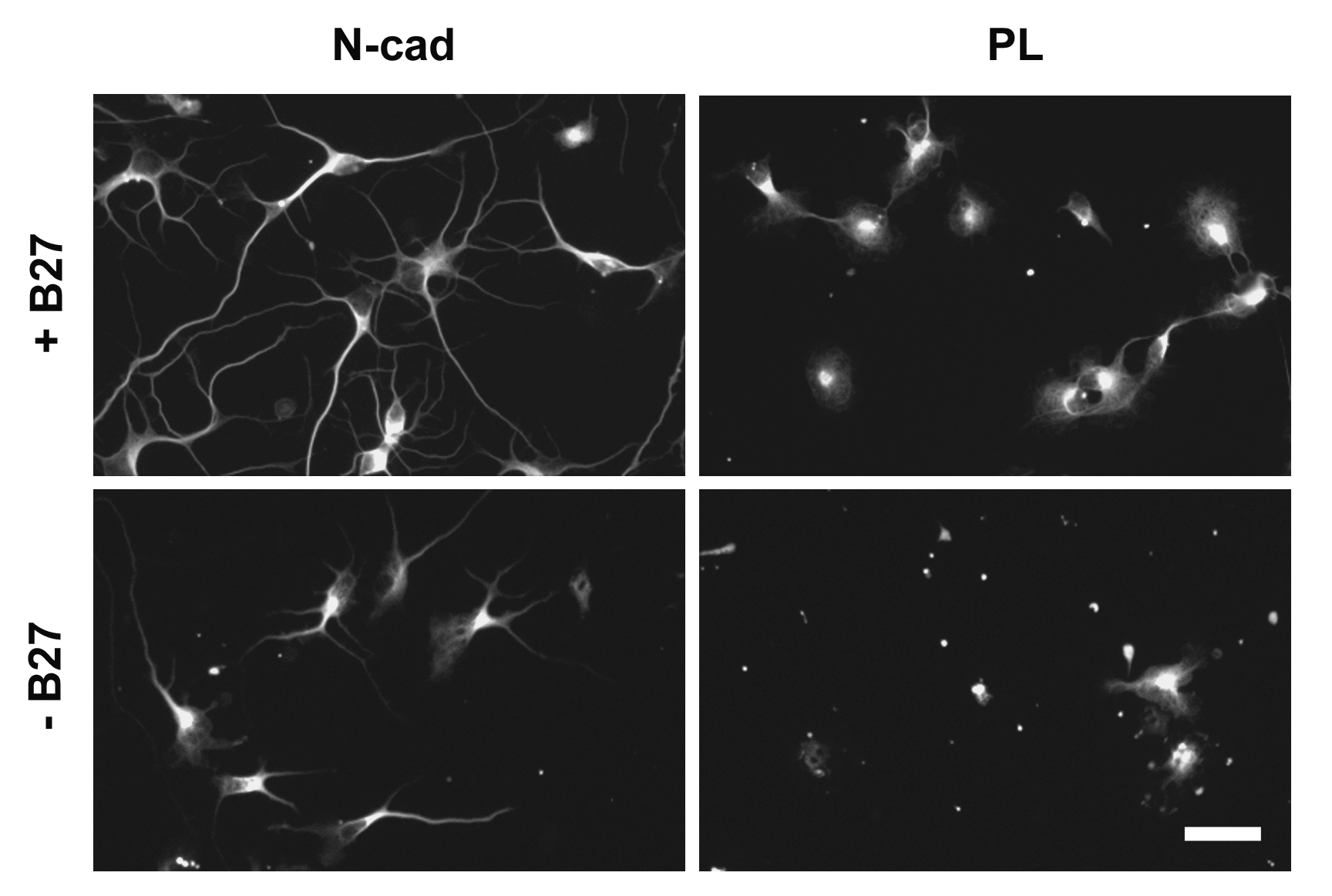

Supplement: Figure S1 — Culture of primary neurons in serum free non supplemented medium affects cell survival. E18.5 rat hippocampus neurons were cultured in MEM medium in the presence of B27 additive and serum (+B27) either on PL (poly-L-lysine) or N-cad (immobilized Ncad-Fc). Alternatively they were cultured on the same types of substrate but in the absence of serum and B27 (−B27). After 24 hours, cells were fixed and stained with anti-βIII tubulin antibody to identify neurons. Although neurons grew well on PL as well as on N-cad in the presence of B27, they died on PL in the absence of the additive and serum. In addition, neurite extension was greatly increased on N-cad compared to PL even in the presence of B27 as expected from previous reports. Similar observations were made with cells cultured from 12.5 mouse ventral spinal cords (not shown). Scale bar: 20 µm. (TIF) [file pone.0033206.s001.tif]

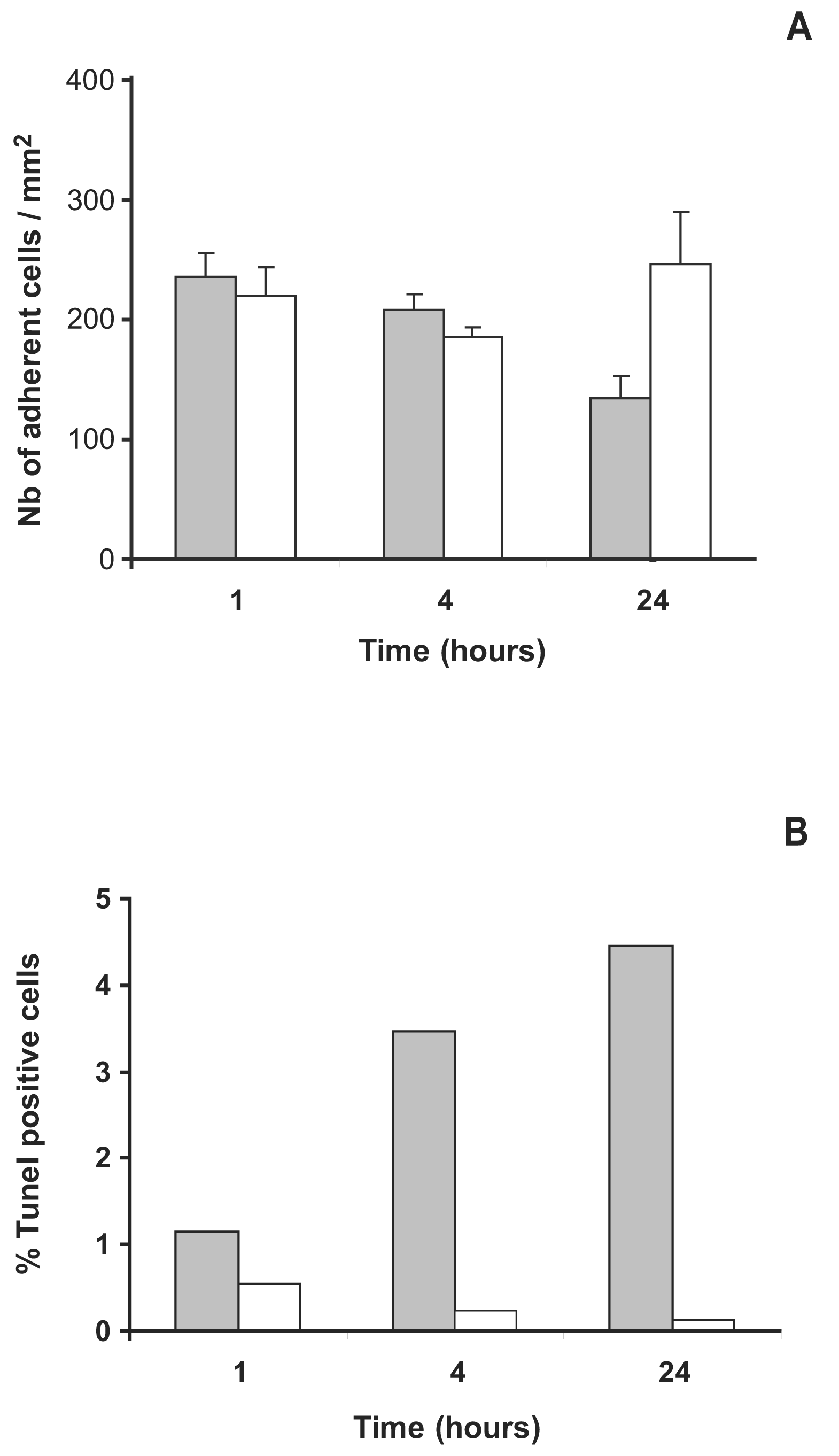

Supplement: Figure S2 — Compared adhesion/survival of primary hippocampal neurons on PL versus Ncad-Fc substrates. E18.5 rat hippocampus neurons were cultured in MEM medium either on PL or N-cad in the absence of serum and B27 additive as reported in Figure 1A. Cultures were fixed at 1, 4 and 24 hours post-seeding and adherent cells were counted (A). Alternatively, preparations were processed for TUNEL labeling and the percentage of TUNEL positive cells determined (B). (TIF) [file pone.0033206.s002.tif]

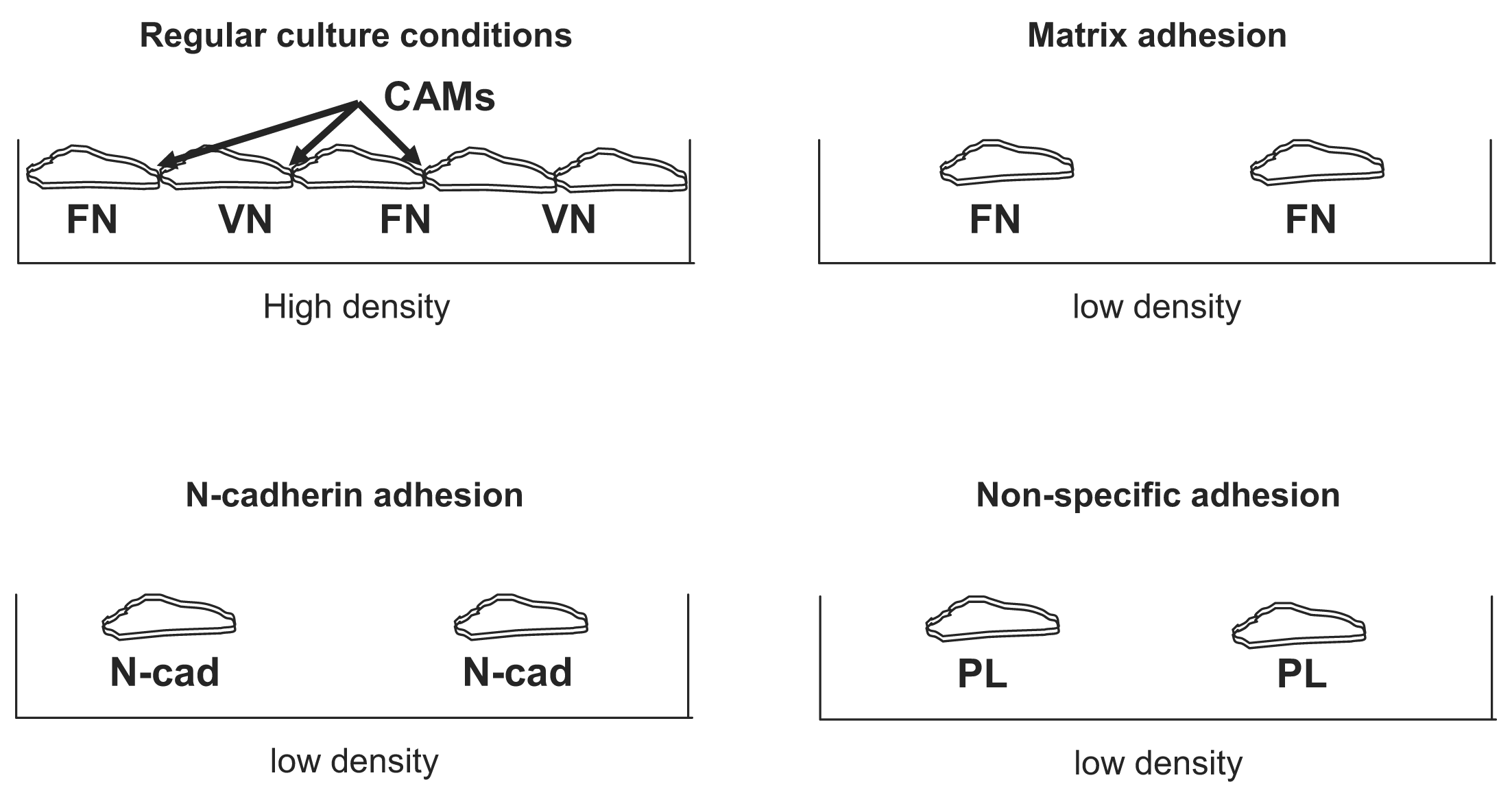

Supplement: Figure S3 — Experimental set up for controlled N-cadherin engagement. In regular cell culture conditions (upper left), cells adhere to the substratum via adsorbed fibronectin (FN) and vitronectin (VN) provided by the serum (cell matrix adhesion) and adhere to each other via cadherins and other cell adhesion molecules (CAMs, cell-cell adhesion). To specifically activate either N-cadherin or fibronectin, GT1-7 cells were plated at low density on N-cad or FN, respectively. To deprive cells of specific cell adhesion, they were seeded on PL which mediates electrostatic cell-surface adhesion. (TIF) [file pone.0033206.s003.tif]

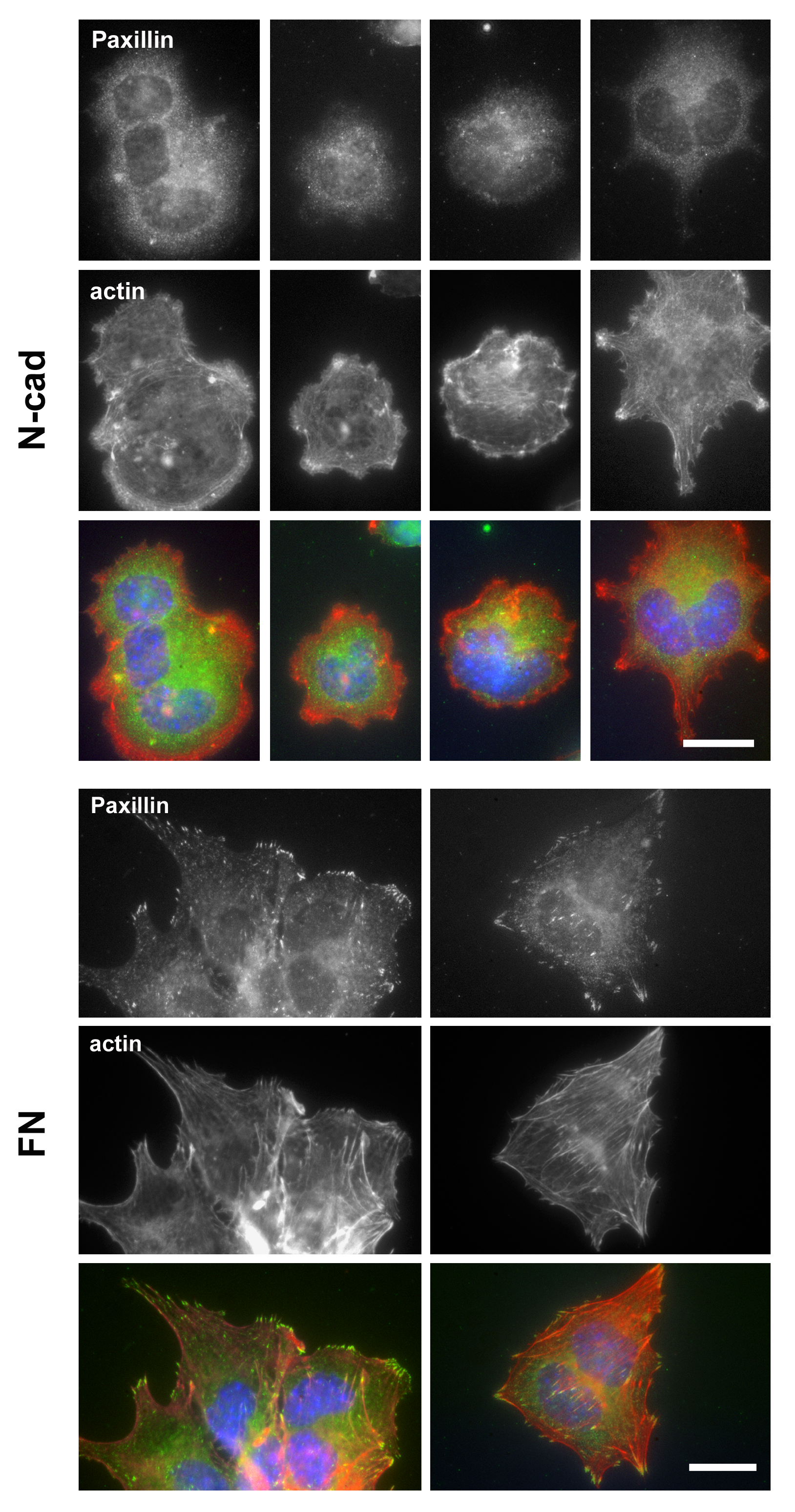

Supplement: Figure S4 — Spreading of cells on N-cad does not mobilize the integrin pathway. GT1-7 cells were cultured on N-cad for 4 hours, then fixed and fluorescently stained for F-actin with phalloïdin (red) and with anti-paxillin antibodies (green). Paxillin staining remained diffuse in the cytoplasm of these cells indicating that integrins were not mobilized on this substrate. In contrast, when GT1-7 cells were cultured on fibronectin (FN) for 4 hours, they displayed expected stress fibers and focal adhesions in which both stainings were strongly accumulated, indicated that indeed integrins have been mobilized and activated in focal adhesions in these culture conditions. Scale bars: 20 µm. (TIF) [file pone.0033206.s004.tif]

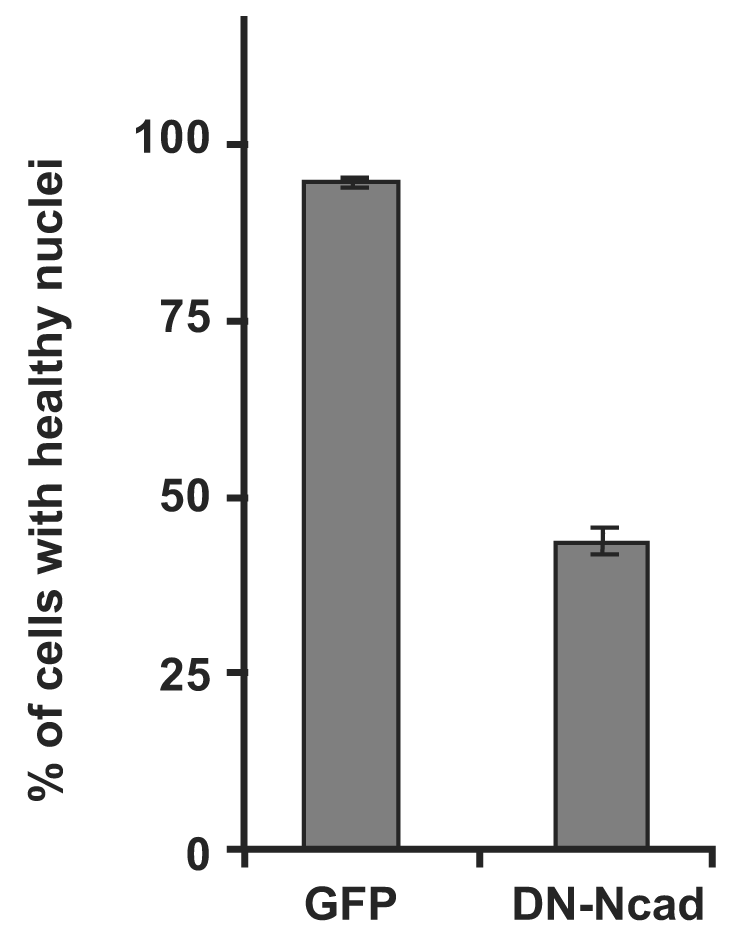

Supplement: Figure S5 — Overexpression of a dominant negative form of N-cadherin drastically impairs the protective effect of N-cad on GT1-7 cells. GT1-7 cells were lipofected with either a dominant negative form of N-cadherin fused to DsRed (DN-Ncad), or GFP alone, starved for 24 hours, then spread on the N-cad substrate. After 24 hours, cells were fixed and processed for nuclei staining. The percentage of transfected cells with normal and condensed nuclei was determined for each condition in three independent experiments and given as mean ± SD. (TIF) [file pone.0033206.s005.tif]

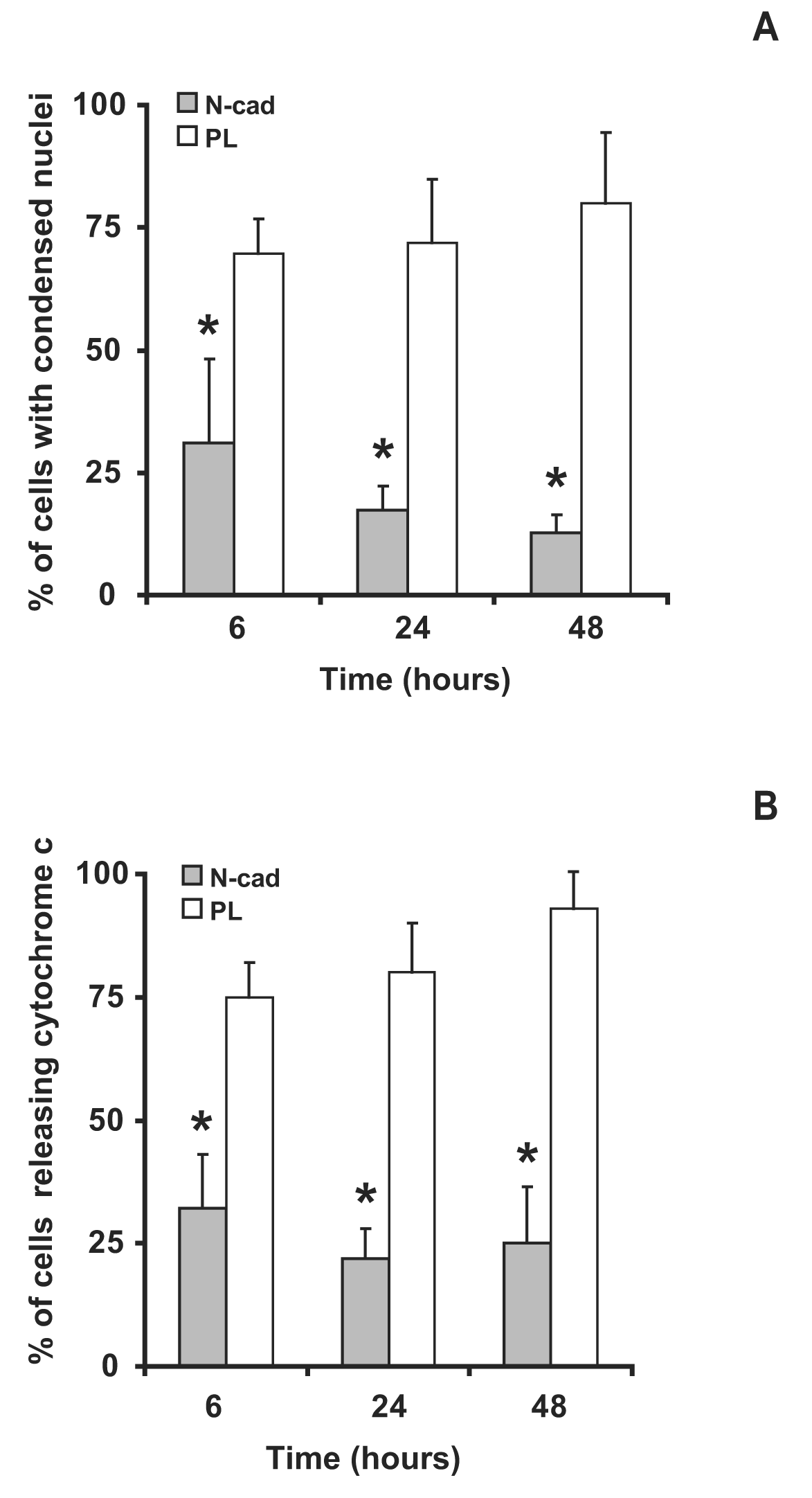

Supplement: Figure S6 — Time course of GT1-7 cell death. Serum-starved cells were seeded at low density on PL or N-cad and grown for the indicated time in serum-free medium. Cell death was measured by nuclear condensation (A) and cytochrome c release (B). Asterisks indicate a significant difference (P<0.05) as compared with PL. (TIF) [file pone.0033206.s006.tif]

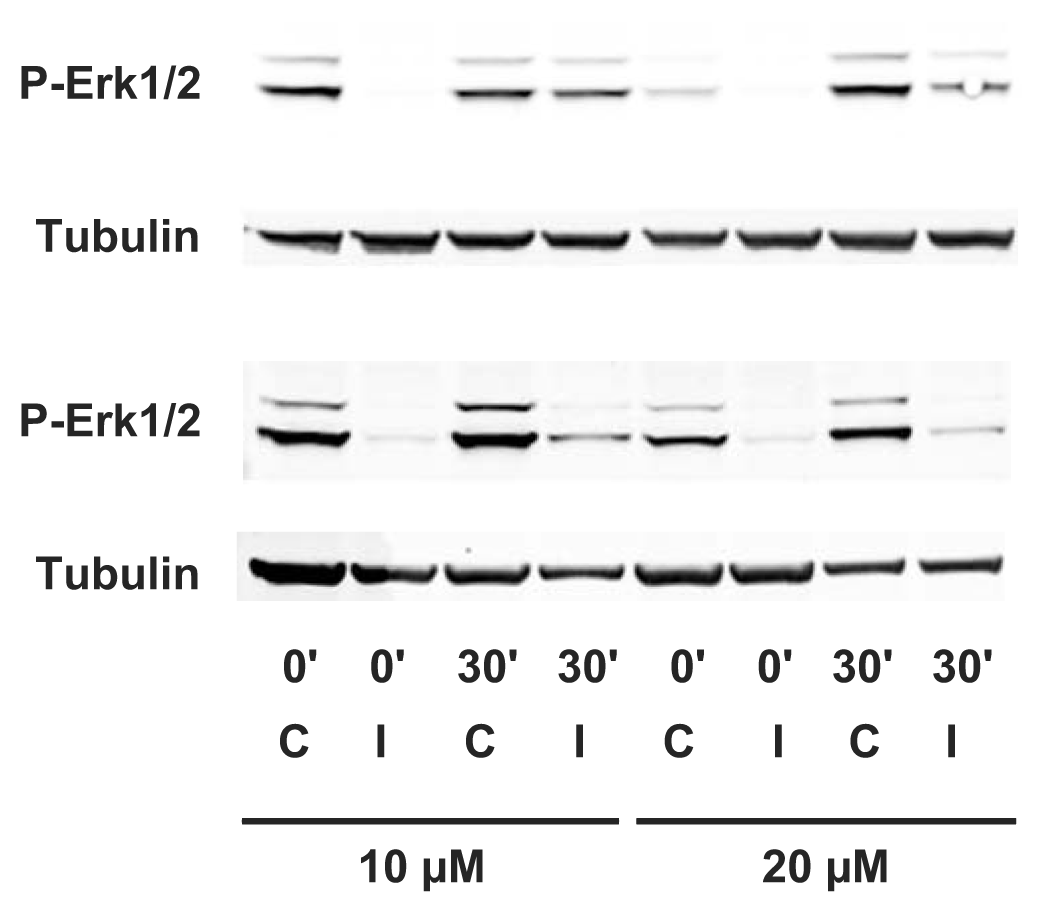

Supplement: Figure S7 — Dose dependent action of U0126 inhibitor. Serum starved GT1-7 cells were treated (I) or not (C) with the U0126 inhibitor. Cultures were then subject to the calcium switch protocol and harvested just after (0′) or 30 minutes (30′) after calcium restoration. Proteins were extracted and analyzed for P-Erk1/2 content by western blotting. The results of two independent experiments are shown. The tubulin content in the extracts was used as a gel loading control. (TIF) [file pone.0033206.s007.tif]

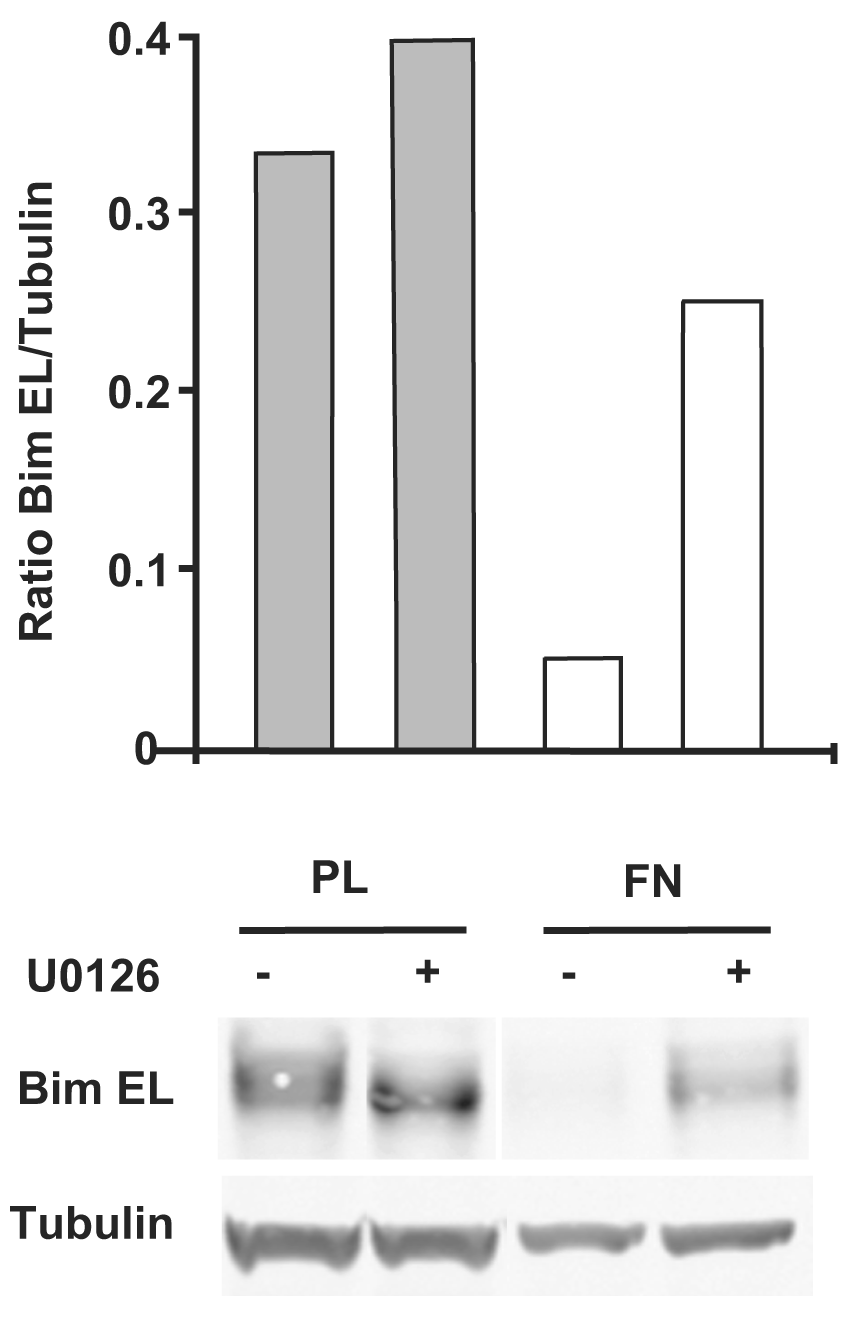

Supplement: Figure S8 — Spreading of GT1-7 cells on fibronectin also down-regulates Bim-EL protein levels. Serum-starved cells were seeded at medium density on PL or FN and incubated with or without 20 µM U0126 for 24 hours. Equal amount of proteins were immunoblotted with either anti-Bim or anti-α-tubulin antibodies. Relative densities of Bim-EL were normalized to α-tubulin. (TIF) [file pone.0033206.s008.tif]
